# Photo- and Cobalt-Catalyzed Cycloisomerization of Unsaturated Guanidines, (Iso-)Ureas, and Carbonates

**DOI:** 10.1021/acs.orglett.4c04695

**Published:** 2025-01-08

**Authors:** Henry Lindner, Erick M. Carreira

**Affiliations:** Laboratory of Organic Chemistry, Department of Chemistry and Applied Biosciences, ETH Zürich, 8093 Zurich, Switzerland

## Abstract

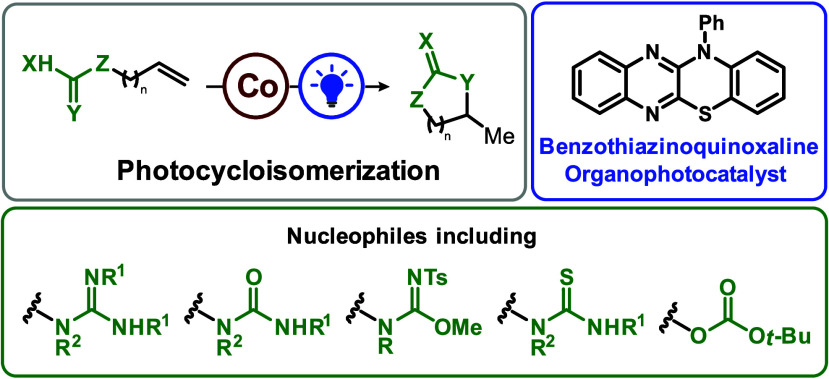

We report intramolecular photocatalyzed cycloisomerization of unactivated olefins with pendant nucleophiles. The reaction proceeds under mild conditions and utilizes guanidines, ureas, isoureas, isothioureas, and carbonates to yield several different five-, six-, and seven-membered heterocycles. Use of benzothiazinoquinoxaline as an organophotocatalyst and cobalt–salen catalyst obviates the need for a stoichiometric oxidant or reductant.

Saturated heterocycles, such as cyclic guanidines, ureas, isoureas, isothioureas, and carbonates, are structural motifs found in natural products, pharmaceuticals, and agrochemicals.^1^ Consequently, the development of efficient methods for their preparation is desirable in organic synthesis. Synthetic approaches for their formation include intramolecular substitutions,^2^ halocyclizations,^3^ and transition-metal-catalyzed reactions.^4^ Herein, we report the photo- and cobalt-catalyzed cycloisomerization of unactivated olefins as a method to access cyclic guanidines as well as ureas, isoureas, isothioureas, and carbonates (Figure 1). The reaction requires only catalytic amounts of benzothiazinoquinoxaline organophotocatalyst (**PC**), Co–salen (**Co-1**), and hydrotriflate salt (Lut-HOTf). Consequently, our photocatalytic approach toward this isohypsic transformation circumvents the need for stoichiometric reductants or oxidants, offering a practical and efficient strategy for the synthesis of heterocycles.

**Figure 1:**
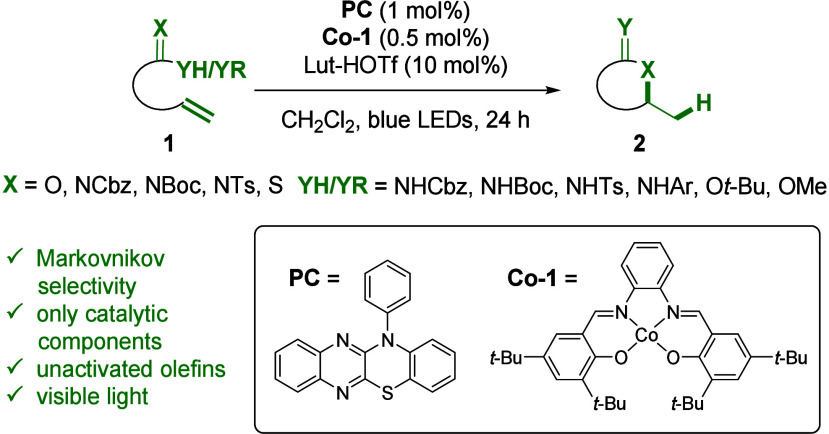
Cobalt- and light-mediated cycloisomerization of unactivated alkenes forms heterocycles with Markovnikov selectivity.

Pioneering contributions by Drago and Mukaiyama have laid the groundwork for the development of transition-metal-catalyzed hydrogen-atom transfer reactions (TM-HAT).^5^ Complexes of iron, manganese, and cobalt have since been shown to effectively functionalize unactivated olefins with radical acceptors displaying Markovnikov selectivity.^6^ Only recently, Shigehisa demonstrated the utility of a cobalt–salen catalyst in combination with *N*-fluorocollidinium salts for the reaction of alkenes with pendant nucleophiles, yielding a variety of heterocycles, such as cyclic ethers and thioethers, pyrrolidines, or guanidines (Figure 2).^7^ The transformation required superstoichiometric amounts of both the chemical oxidant and hydrosilane as a reductant to adjust the oxidation state of the cobalt catalyst in line with the overall paradigm set out by Drago and Mukaiyama. Zhu later developed cycloisomerization conditions that were able to utilize molecular oxygen, while excess silane was still required.^8^ Zhu also introduced an electrochemical approach for cobalt-catalyzed cycloisomerizations of alkenes, which explored anodic oxidation as an alternative to chemical oxidants.^9^ While these strategies represent significant progress, the fundamental transformations are redox-neutral or isohypsic, and thus, further development of methods that minimize the use of stoichiometric oxidants and reductants remains a priority for enhancing sustainability and efficiency in synthetic chemistry.

**Figure 2:**
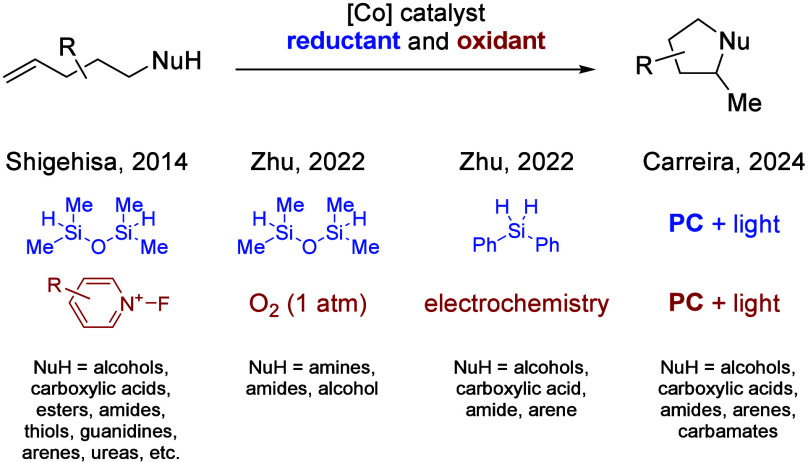
Previous cobalt-catalyzed approaches toward cycloisomerization reactions.

We previously demonstrated the photocycloisomerization of unactivated olefins using a range of *N*-, *O*-, and *C*-nucleophiles to yield, among others, pyrrolidines, pyrimidines, tetrahydrofurans, and tetrahydropyrans.^10^ Given our long-standing interest in the functionalization of alkenes,^11^ we consequently sought to extend this methodology to access an even broader array of heterocycles. In a first experiment (entry a in Table 1), allylic guanidine **1a** was subjected to 1 mol % **PC**, 0.5 mol % **Co-1**, and 10 mol % Lut-HOTf and irradiated for 24 h with blue light-emitting diodes (LEDs) (for details on the photoreactor set-up, see the Supporting Information). Gratifyingly, the reaction yielded the cyclized product **2a** in 92% yield.

**Table 1:**
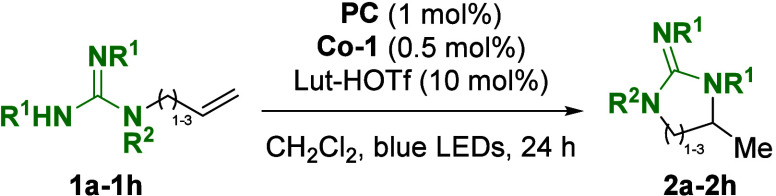

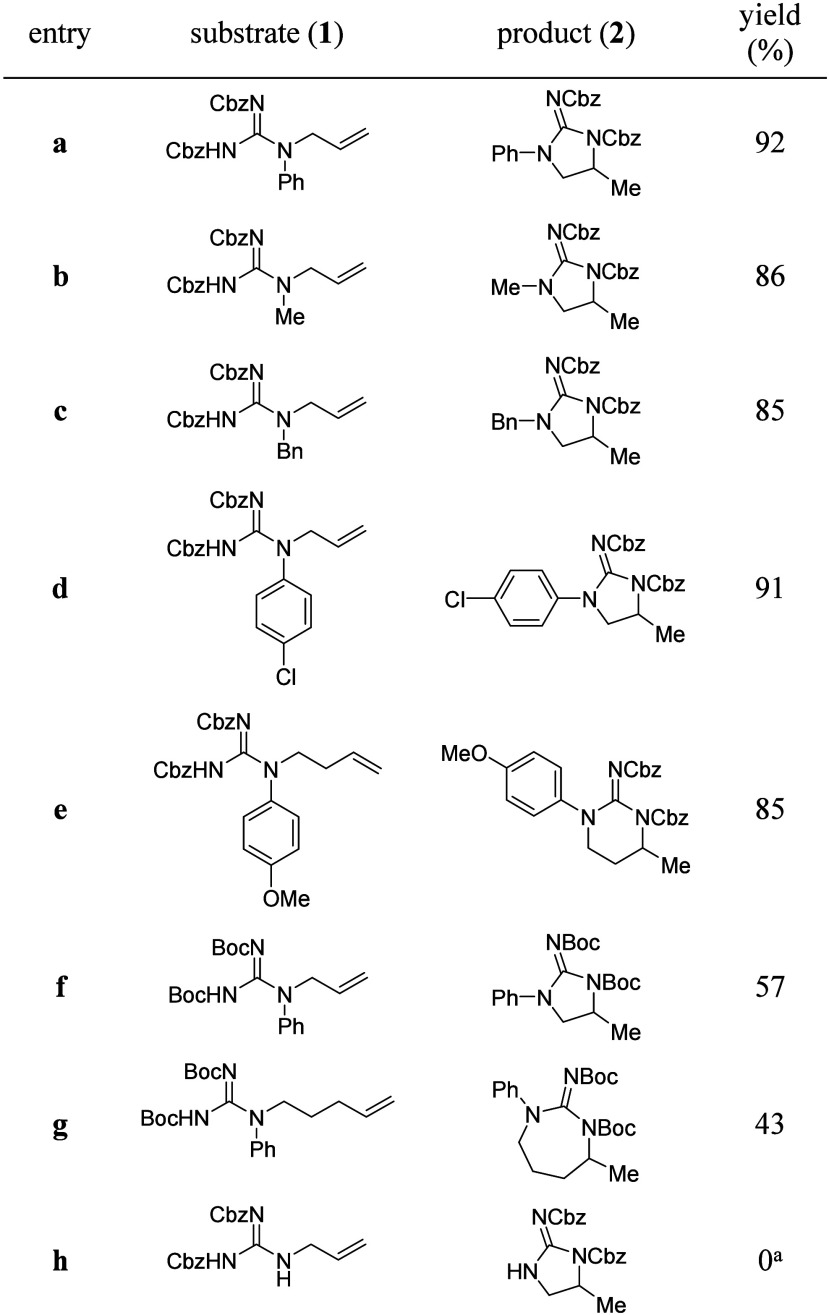
Substrate Scope for the Photocycloisomerization Yielding Cyclic Guanidines

a Yield determined by analysis of ^1^H NMR of the unpurified reaction mixture with mesitylene as the internal standard.

With these promising results in hand, we investigated the generality and scope of the reaction. The initial focus remained on the use of protected guanidines as nucleophiles (Table 1). Alkyl-substituted guanidines **1b** and **1c** readily cyclized under standard conditions to give the corresponding products in 86 and 85% yields, respectively. The reaction proceeded equally well with substrates containing substituted anilines to give products **2d** and **2e** in 91 and 85% yields, respectively. To test the feasibility of different protecting groups, *N*-Boc-protected guanidines were subjected to the reaction and furnished products **2f** and **2g** in 43 and 57% yields. Notably, the conditions were also able to affect cyclization, leading to the formation of the corresponding seven-membered ring. Unsubstituted guanidine **1h** did not react under standard conditions, and the starting material was reisolated.

Next, we sought to examine the scope of ureas as nucleophiles (Table 2). A variety of isoureas (**2i**–**2k**) were accessed in 76–93% yields. Notably, the reaction tolerated the use of 1,1-disubstituted olefins, and compound **1l** was converted to product in 88% yield. When the method was extended toward Ts-protected starting materials, product **2m** was accessed in 56% yield. The photocycloisomerization of thiourea **1n** furnished isothiourea **2n** in 83% yield.

**Table 2:**
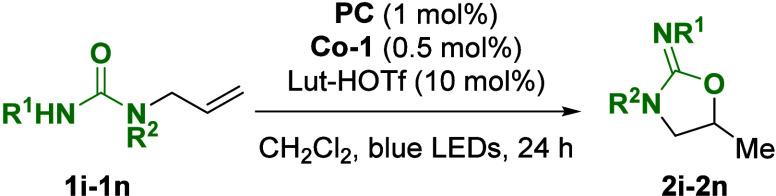

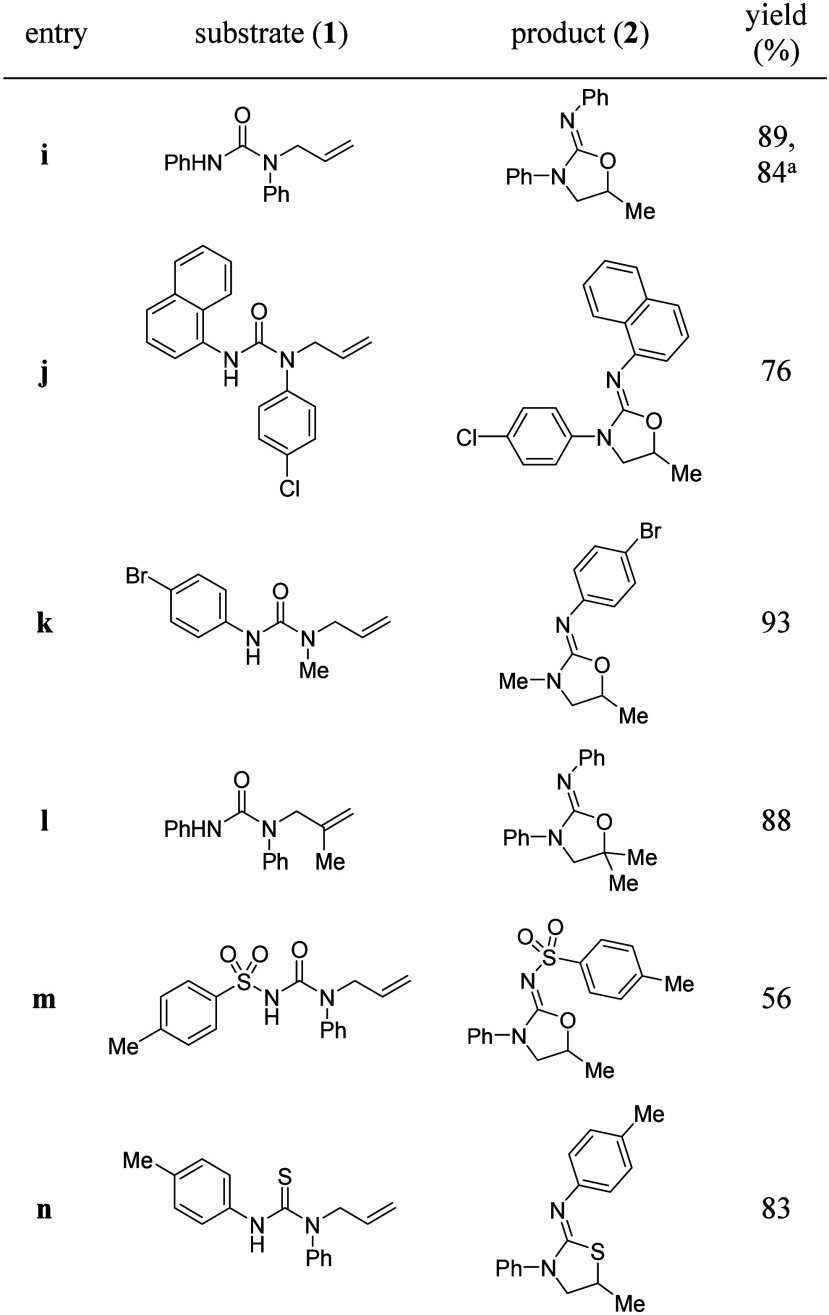
Substrate Scope for the Photocycloisomerization Yielding Cyclic Isoureas and Isothioureas

a Reaction conducted on a 3 mmol scale.

When *tert*-butyl carbonates **1o**–**1q** were subjected to the reaction conditions, the formation of the corresponding cyclic carbonates was observed in 46–84% yield (Figure 3). This transformation is proposed to proceed by cyclization and subsequent isobutylene extrusion from an oxonium intermediate. To synthesize cyclic ureas, substrate **1r** was prepared and yielded 13% of the desired urea under the standard conditions. Exchange of the solvent with an acetone–water (20:1) mixture led to improved yield, and product **2r** was isolated in 55% yield.

**Figure 3:**
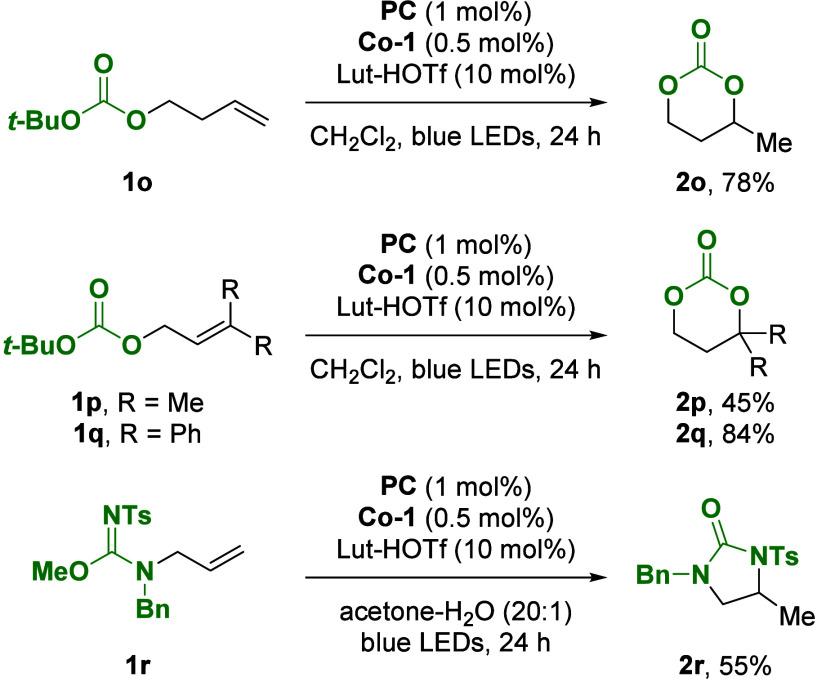
Substrate scope for the photocycloisomerization yielding cyclic carbonates and ureas.

On the basis of the observed reactivity and prior work,^10a,10,12^ a mechanistic construct for the reported photocycloisomerization reaction is proposed (Figure 4). Reduction of the [Co^II^]–salen catalyst by the excited-state photocatalyst forms an anionic [Co^I^] complex. Subsequent reversible protonation furnishes the conjugate acid [Co^III^]–H, which can transfer a hydrogen atom to an unactivated olefin.^6^ The resulting intermediate is oxidized by the oxidized ground-state photocatalyst to yield a [Co^IV^]–alkyl species.^13^ Base-mediated, intramolecular substitution of the [Co] complex with a pendant nucleophile leads to product formation, while the [Co^II^] catalyst is regenerated.

**Figure 4:**
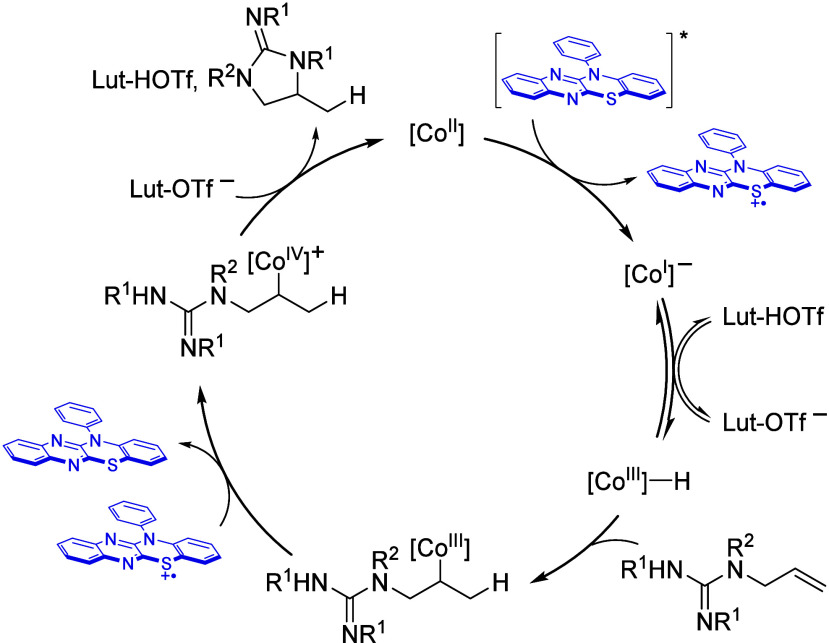
Proposed working catalytic cycle.

In conclusion, we have expanded on our versatile method for the Markovnikov-selective photocycloisomerization of unactivated alkenes to enable the efficient synthesis of cyclic guanidines, isoureas, isothioureas, ureas, and carbonates. The reaction is characterized by mild and user-friendly conditions and operates without the requirement of stoichiometric reductants or oxidants. Consequently, this work highlights the utility of the [Co^I^]^−^ ⇌ [Co^III^]–H equilibrium in HAT chemistry and represents a valuable example for the combination of organophotoredox and base metal catalysis to advance the development of synthetic methods. More broadly, photochemistry provides new ways to perform isohypsic reactions without the use of superfluous redox reagents.

## Data Availability

The data underlying this study are available in the published article and its Supporting Information.

## Supporting Information Available

Experimental procedures and characterization data for all new compounds (PDF)
