# Supplementary material for: Photo- and Cobalt-Catalyzed Cycloisomerization of Unsaturated Guanidines, (Iso-)Ureas, and Carbonates
Source: Org Lett. 2025 Jan 8;27(2):704–8. doi: 10.1021/acs.orglett.4c04695 (PMC11744792; doi:10.1021/acs.orglett.4c04695)
Supplement: Supplementary file 1 — ol4c04695_si_001.pdf [file ol4c04695_si_001.pdf]

Supporting Information

Photo- and Cobalt-Catalyzed Cycloisomerization of Unsaturated Guanidines, (Iso-)Ureas, and Carbonates

Henry Lindner and Erick M. Carreira*

ETH Zürich, Department of Chemistry and Applied Biosciences, Laboratory of Organic Chemistry, 8093 Zurich, Switzerland

Table of Contents

1. General Remarks 2
2. General Procedure 4
3. Substrate Scope 5
4. Preparation of Starting Materials 22
5. ^{1}H and ^{13}C NMR Spectra 24
6. References 42

1. General Remarks

Procedure and chemicals

Unless otherwise stated, all reactions were carried out under nitrogen. Reactions in the 350 W photoreactor were performed in 38.2x22 mm crimp-neck vials (Labsolute, Art. Nr. 7615908) that were charged with a magnetic stirrer bar (PTFE, 6x15 mm, Semadeni Plastics Group, Art. 249) and sealed with a crimp cap (infochroma ag, 8087-Bu).

Anhydrous solvents over molecular sieves were purchased from Acros. For flash column chromatography, Sigma-Aldrich silica gel sorbent (high purity grade (9385), 230-400 mesh particle size, pore size 60) was used as a stationary phase. Chemicals were purchased from commercial suppliers and purified where appropriate.

Thin-Layer Chromatography

Analytical thin layer chromatography (TLC) was performed on glass plates from Supelco® (TLC silica gel 60 F_{254}: 25 glass plates, 20 x 20 cm) and visualized via exposure to ultraviolet light (254 nm or 365 nm) or TLC stain (aqueous potassium permanganate solution or aqueous ceric ammonium molybdate solution followed by heating).

Nuclear Magnetic Resonance Spectroscopy

All NMR spectra were measured in deuterated solvents at room temperature with a Bruker Avance 400 (400 MHz, equipped with 9.4 T magnet and BBFO probe), Bruker Ascend 400 (400 MHz, equipped with 9.4 T magnet and BBFO probe), Bruker Ultrashield 400 (400 MHz, equipped with 9.4 T magnet and BBFO probe), or a Oxford 400 (400 MHz, equipped with 9.4 T magnet and BBFO probe). The chemical shifts are referenced to the solvent residual signal ($CDCl_3$, ^{1}H: δ = 7.26 ppm, ^{13}C: δ = 77.16 ppm) and reported in parts per million (ppm). The following abbreviations are used in reporting NMR data: s = singlet, d = doublet, t = triplet, q = quartet, b = broad, dd = doublet of doublets, m = multiplet, etc.

High-Resolution Mass Spectrometry

High resolution mass spectrometric data were obtained by the mass spectrometry service of the Laboratory of Organic Chemistry at ETH Zurich on a Bruker Daltonics maXis ESI-QTOF or a Bruker Daltonics maXis II ESI-QTOF or a Thermo Q-Exactive GC Orbitrap instrument and are reported as (*m/z*).

IR Spectroscopy

Infrared spectra were recorded on a Perkin Elmer Two FT-IR spectrometer as thin films. Absorptions are given in wavenumbers (cm^{-1}).

Photoreaction Set-Up[1,2]

All photoreactions were carried out in a custom-designed photoreactor. It features ten circularly arranged 35 W blue LEDs (manufacturer: Luminus, model: SBR-70-B-R75-KG300), mounted on copper heat sinks, which surround the central reaction vessel holder. The distance from the light source to the irradiation vessel was approx. 2 cm. The emission spectrum of the blue LED reactor shows a maximum intensity at a wavelength of λ_{max} = 446 nm with FWHM = 20 nm.

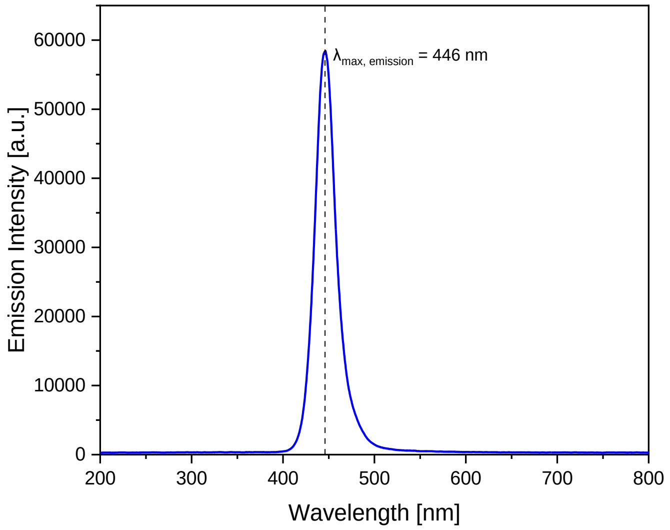

Figure 1. UV-vis emission spectrum of the blue LED photoreactor. The figure is taken from.[2]

2. General Procedure

GP1: Photo-Cycloisomerization

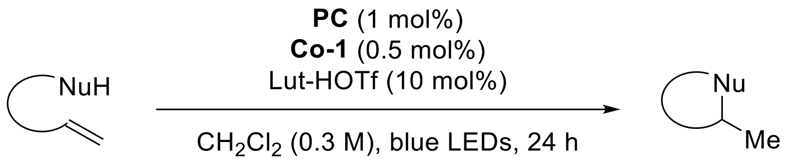

A glass crimp vial was charged with **Co-1** (0.90°mg, 0.0015°mmol, 0.50°mol%), **PC** (0.98 mg, 0.0030 mmol, 1.0 mol%), lutidinium triflate (Lut-HOTf, 7.7 mg, 0.030 mmol, 10 mol%), starting material (0.30 mmol, 1.0 equiv), and a magnetic stirring bar. The vial was capped with a crimp cap and evacuated/backfilled with nitrogen two times. After addition of anhydrous CH_2Cl_2 (1.0 mL, 0.30 M), the reaction mixture was degassed by bubbling nitrogen for 2 min. The reaction was irradiated in a 350 W photoreactor (*vide supra*) for 24 h. The solvent was removed *in vacuo* and purification using silica gel chromatography with the appropriate eluent was employed to obtain pure product.

Note: The photo-cycloisomerization can also be conducted by using a stock solution. In this case, the appropriate amounts of **Co-1** (0.90°mg, 0.0015°mmol, 0.50°mol%), **PC** (0.98 mg, 0.0030 mmol, 1.0 mol%), lutidinium triflate (7.7 mg, 0.030 mmol, 10.0 mol%) were dissolved in 1 mL anhydrous, degassed CH_2Cl_2 and added to the already purged crimp vial containing the starting material. The observed yields are comparable for both procedures.

3. Substrate Scope

Compound 2a:

Benzyl 2-(((benzyloxy)carbonyl)imino)-5-methyl-3-phenylimidazolidine-1-carboxylate

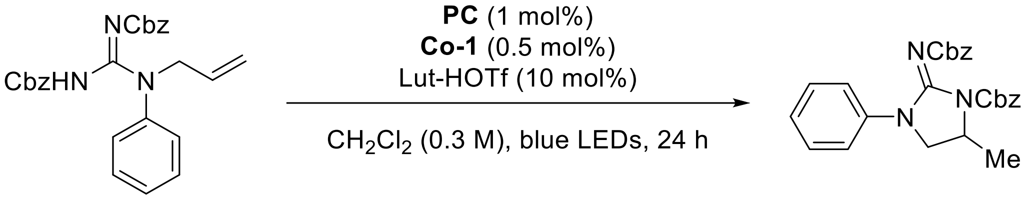

Guanidine **2a** was prepared via GP1 from alkene **1a** (133 mg, 0.300 mmol, 1.00 equiv) in CH_2Cl_2. The crude product was purified via flash column chromatography (10-50% Et_2O in pentane) to give **2a** as a white solid.

Yield: 122 mg, 0.275 mmol, 92%

^{1}H NMR (400 MHz, $CDCl_3$): δ (ppm) = 7.50 – 7.45 (m, 2H), 7.39 – 7.24 (m, 12H), 7.18 – 7.13 (m, 1H), 5.21 (d, *J* = 12.2 Hz, 1H), 5.09 (d, *J* = 12.2 Hz, 1H), 5.06 – 4.96 (m, 2H), 4.49 (dqd, *J* = 8.2, 6.4, 1.8 Hz, 1H), 4.17 (dd, *J* = 9.5, 8.1 Hz, 1H), 3.38 (dd, *J* = 9.5, 1.9 Hz, 1H), 1.46 (d, *J* = 6.4 Hz, 3H).

^{13}C NMR (101 MHz, $CDCl_3$): δ (ppm) = 159.7, 151.4, 148.2, 139.0, 137.0, 135.2, 129.1, 128.7, 128.6, 128.6, 128.3, 128.3, 127.8, 125.6, 122.1, 68.5, 67.5, 52.8, 51.5, 20.5.

IR (thin film, cm^{-1}): 3033, 2965, 1752, 1714, 1685, 1625, 1593, 1499, 1456, 1425, 1390, 1343, 1275, 1217, 1141, 1073, 1027, 757, 697.

HRMS (ESI+): *m/z* for $C_{26}H_{26}N_3O_4$ $[M+H]^+$: calc.: 444.1918, found: 444.1911.

TLC: R_f = 0.45 (SiO_2, 50% EtOAc in hexane).

Compound 2b:

Benzyl 2-(((benzyloxy)carbonyl)imino)-3,5-dimethylimidazolidine-1-carboxylate

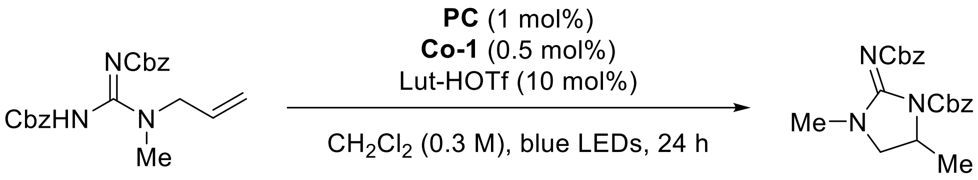

Guanidine **2b** was prepared via GP1 from alkene **1b** (114 mg, 0.300 mmol, 1.00 equiv) in CH_2Cl_2. The crude product was purified via flash column chromatography (20-100% Et_2O in pentane) to give **2b** as a pale-yellow oil.

Yield: 98.0 mg, 0.257 mmol, 86%

^{1}H NMR (400 MHz, $CDCl_3$): δ (ppm) = 7.38 – 7.21 (m, 10H), 5.15 (d, *J* = 12.2 Hz, 1H), 5.08 – 4.95 (m, 3H), 4.36 (dqd, *J* = 8.7, 6.4, 2.3 Hz, 1H), 3.67 (dd, *J* = 9.7, 8.4 Hz, 1H), 3.00 – 2.91 (m, 4H), 1.38 (d, *J* = 6.4 Hz, 3H).

^{13}C NMR (101 MHz, $CDCl_3$): δ (ppm) = 159.8, 151.6, 151.4, 137.2, 135.2, 128.6, 128.5, 128.5, 128.3, 128.3, 127.7, 68.3, 67.2, 52.9, 51.7, 32.3, 20.7.

IR (thin film, cm^{-1}): 2932, 2878, 1747, 1712, 1677, 1616, 1496, 1454, 1389, 1360, 1342, 1267, 1213, 1175, 1136, 1063, 1045, 974, 911, 727, 697, 644.

HRMS (ESI+): *m/z* for $C_{21}H_{24}N_3O_4$ $[M+H]^+$: calc.: 382.1761, found: 382.1760.

TLC: R_f = 0.40 (SiO_2, EtOAc).

Compound 2c:

Benzyl 3-benzyl-2-(((benzyloxy)carbonyl)imino)-5-methylimidazolidine-1-carboxylate

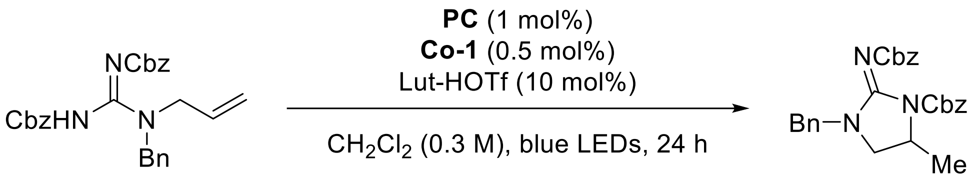

Guanidine **2c** was prepared via GP1 from alkene **1c** (137 mg, 0.300 mmol, 1.00 equiv) in CH_2Cl_2. The crude product was purified via flash column chromatography (10-100% Et_2O in pentane) to give **2c** as a pale-yellow oil.

Yield: 117 mg, 0.256 mmol, 85%

^{1}H NMR (400 MHz, $CDCl_3$): δ (ppm) = 7.40 – 7.23 (m, 15H), 5.17 (d, *J* = 12.2 Hz, 1H), 5.12 – 4.99 (m, 3H), 4.74 (d, *J* = 14.8 Hz, 1H), 4.43 – 4.29 (m, 2H), 3.50 (dd, *J* = 9.7, 8.4 Hz, 1H), 2.81 (dd, *J* = 9.7, 2.2 Hz, 1H), 1.30 (d, *J* = 6.4 Hz, 3H).

^{13}C NMR (101 MHz, $CDCl_3$): δ (ppm) = 160.0, 151.7, 151.5, 137.3, 135.4, 135.3, 129.0, 128.7, 128.6, 128.4, 128.4, 128.3, 128.1, 127.7, 68.4, 67.4, 51.8, 49.8, 48.8, 20.7.

IR (thin film, cm^{-1}): 3032, 1750, 1716, 1680, 1615, 1496, 1454, 1392, 1341, 1264, 1183, 1129, 1078, 1028, 754, 698.

HRMS (ESI+): *m/z* for $C_{27}H_{28}N_3O_4$ $[M+H]^+$: calc.: 458.2074, found: 458.2071.

TLC: R_f = 0.24 (SiO_2, 50% EtOAc in hexane).

Compound 2d:

Benzyl 2-(((benzyloxy)carbonyl)imino)-3-(4-chlorophenyl)-5-methylimidazolidine-1-carboxylate

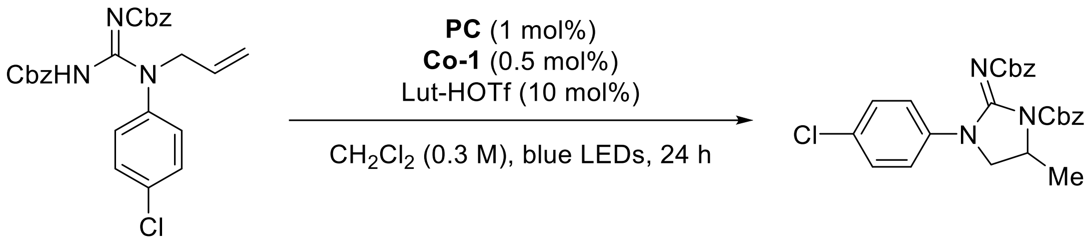

Guanidine **2d** was prepared via GP1 from alkene **1d** (143 mg, 0.300 mmol, 1.00 equiv) in CH_2Cl_2. The crude product was purified via flash column chromatography (10-60% Et_2O in pentane) to give **2d** as an off-white solid.

Yield: 130 mg, 0.272 mmol, 91%

^{1}H NMR (400 MHz, $CDCl_3$): δ (ppm) = 7.47 – 7.41 (m, 2H), 7.39 – 7.24 (m, 12H), 5.20 (d, *J* = 12.1 Hz, 1H), 5.13 – 4.96 (m, 3H), 4.49 (dqd, *J* = 8.2, 6.4, 1.8 Hz, 1H), 4.13 (dd, *J* = 9.4, 8.1 Hz, 1H), 3.35 (dd, *J* = 9.3, 1.9 Hz, 1H), 1.46 (d, *J* = 6.4 Hz, 3H).

^{13}C NMR (101 MHz, $CDCl_3$): δ (ppm) = 159.5, 151.3, 147.8, 137.6, 136.9, 135.1, 130.8, 129.1, 128.8, 128.7, 128.6, 128.4, 128.4, 127.9, 123.1, 68.6, 67.6, 52.5, 51.4, 20.6.

IR (thin film, cm^{-1}): 2967, 2887, 1753, 1624, 1589, 1496, 1435, 1391, 1344, 1277, 1219, 1141, 1072, 1009, 756, 698.

HRMS (ESI+): *m/z* for $C_{26}H_{25}ClN_3O_4$ $[M+H]^+$: calc.: 478.1528, found: 478.1519.

TLC: R_f = 0.42 (SiO_2, 50% EtOAc in hexane).

Compound 2e:

Benzyl 2-(((benzyloxy)carbonyl)imino)-3-(4-methoxyphenyl)-6-methyltetrahydropyrimidine-1(2H)-carboxylate

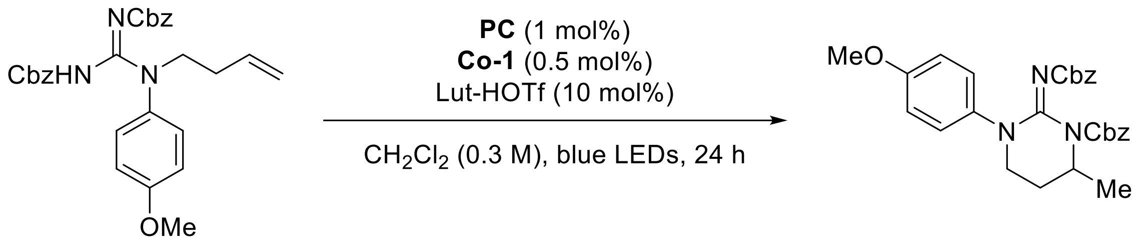

Guanidine **2e** was prepared via GP1 from alkene **1e** (146 mg, 0.300 mmol, 1.00 equiv) in CH_2Cl_2. The crude product was purified via flash column chromatography (20-100% Et_2O in pentane) to give **2e** as a pale-yellow oil.

Yield: 125 mg, 0.256 mmol, 85%

^{1}H NMR (400 MHz, $CDCl_3$): δ (ppm) = 7.36 – 7.21 (m, 10H), 7.20 – 7.15 (m, 2H), 6.90 – 6.84 (m, 2H), 5.17 (d, *J* = 12.3 Hz, 1H), 4.90 (d, *J* = 2.6 Hz, 2H), 4.79 (d, *J* = 12.3 Hz, 1H), 4.57 (dp, *J* = 9.2, 6.6 Hz, 1H), 3.79 (s, 3H), 3.70 (ddd, *J* = 12.8, 11.5, 3.9 Hz, 1H), 3.44 (ddd, *J* = 12.8, 5.3, 3.3 Hz, 1H), 2.41 (ddt, *J* = 12.9, 9.1, 3.6 Hz, 1H), 1.90 – 1.76 (m, 1H), 1.46 (d, *J* = 6.5 Hz, 3H).

^{13}C NMR (101 MHz, $CDCl_3$): δ (ppm) = 160.6, 158.3, 153.9, 153.7, 137.0, 135.7, 135.6, 128.8, 128.6, 128.5, 128.3, 128.3, 127.8, 127.2, 114.6, 68.5, 67.2, 55.6, 51.0, 48.7, 33.4, 22.3.

IR (thin film, cm^{-1}): 2956, 2837, 1824, 1675, 1612, 1578, 1510, 1454, 1389, 1285, 1248, 1225, 1156, 1029, 1002, 912, 831, 726, 698.

HRMS (ESI+): *m/z* for $C_{28}H_{30}N_3O_5$ $[M+H]^+$: calc.: 488.2180, found: 488.2172.

TLC: R_f = 0.31 (SiO_2, 50% EtOAc in hexane).

Compound 2f:

***tert*-Butyl 2-((tert-butoxycarbonyl)imino)-5-methyl-3-phenylimidazolidine-1-carboxylate**

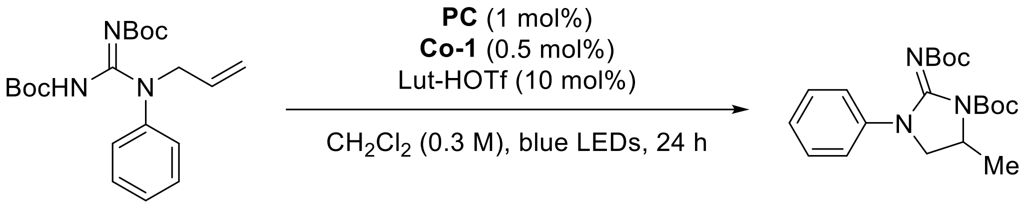

Guanidine **2f** was prepared via GP1 from alkene **1f** (113 mg, 0.300 mmol, 1.00 equiv) in CH_2Cl_2. The crude product was purified via flash column chromatography (10-50% Et_2O in pentane) to give **2f** as a white solid.

Yield: 64.2°mg, 0.171°mmol, 57%

^{1}H NMR (400 MHz, $CDCl_3$): δ (ppm) = 7.51 – 7.46 (m, 2H), 7.37 – 7.29 (m, 2H), 7.15 – 7.08 (m, 1H), 4.36 (dqd, *J* = 8.2, 6.4, 1.8 Hz, 1H), 4.13 (dd, *J* = 9.3, 8.1 Hz, 1H), 3.33 (dd, *J* = 9.3, 1.9 Hz, 1H), 1.52 (s, 9H), 1.45 (s, 9H), 1.43 (d, *J* = 6.4 Hz, 3H).

^{13}C NMR (101 MHz, $CDCl_3$): δ (ppm) = 159.1, 150.0, 148.2, 139.8, 129.1, 125.0, 122.0, 82.7, 79.0, 52.9, 51.4, 28.4, 28.3, 20.7.

IR (thin film, cm^{-1}): 2977, 2932, 1745, 1683, 1624, 1594, 1502, 1421, 1367, 1345, 1285, 1257, 1229, 1152, 1134, 1078, 910, 759, 692.

HRMS (ESI+): *m/z* for $C_{20}H_{30}N_3O_4$ $[M+H]^+$: calc.: 376.2231, found: 376.2228.

TLC: R_f = 0.45 (SiO_2, 50% EtOAc in hexane).

Compound 2g:

***tert*-Butyl 2-((tert-butoxycarbonyl)imino)-7-methyl-3-phenyl-1,3-diazepane-1-carboxylate**

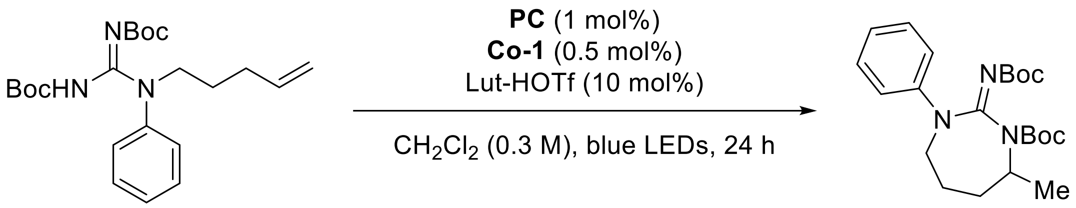

Guanidine **2g** was prepared via GP1 from alkene **1g** (121 mg, 0.300 mmol, 1.00 equiv) in CH_2Cl_2. The crude product was purified via flash column chromatography (5-40% Et_2O in pentane) to give **2g** as a white solid.

Yield: 51.9 mg, 0.129 mmol, 43%

^{1}H NMR (400 MHz, $CDCl_3$): δ (ppm) = 7.39 – 7.28 (m, 4H), 7.22 – 7.15 (m, 1H), 4.57 – 4.44 (m, 1H), 3.80 – 3.68 (m, 1H), 3.67 – 3.55 (m, 1H), 2.00 – 1.86 (m, 2H), 1.72 – 1.58 (m, 2H), 1.54 – 1.36 (m, 21H).

^{13}C NMR (101 MHz, $CDCl_3$): δ (ppm) = 152.1, 144.1, 129.3, 126.5, 126.2, 81.7, 79.5, 53.0, 50.6, 30.9, 28.4, 28.3, 22.5, 17.2.

IR (thin film, cm^{-1}): 2977, 2932, 1713, 1641, 1591, 1495, 1454, 1418, 1390, 1366, 1325, 1304, 1254, 1149, 1117, 1086, 1025, 915, 853, 758, 695.

HRMS (ESI+): *m/z* for $C_{22}H_{34}N_3O_4$ $[M+H]^+$: calc.: 404.2544, found: 404.2537.

TLC: R_f = 0.31 (SiO_2, 20% EtOAc in hexane).

Compound 2i:

5-Methyl-*N*,3-diphenyloxazolidin-2-imine

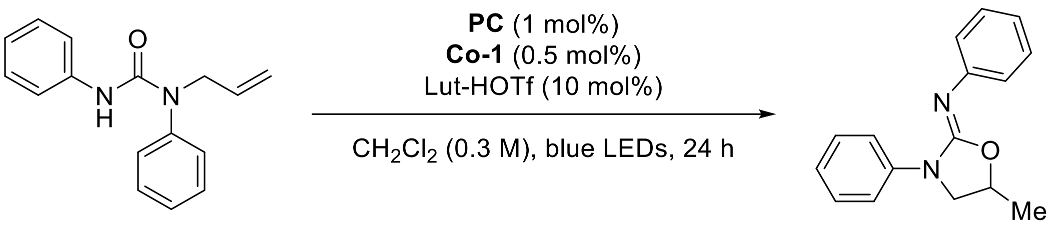

Isourea **2i** was prepared via GP1 from alkene **1i** (75.7 mg, 0.300 mmol, 1.00 equiv) in CH_2Cl_2. The crude product was purified via flash column chromatography (5-20% Et_2O in pentane) to give **2i** as a white solid.

Note: Isourea **2i** was prepared on larger scale via GP1 from alkene **1i** (757 mg, 3.00 mmol, 1.00 equiv) in CH_2Cl_2. The required irradiation time for full conversion was increased to 48 h and the crude product was purified via flash column chromatography (5-20% Et_2O in pentane) to give **2i** as a white solid (634 mg, 2.51 mmol) in 84% yield.

Yield: 67.2 mg, 0.266 mmol, 89%

^{1}H NMR (400 MHz, $CDCl_3$): δ (ppm) = 7.82 – 7.78 (m, 2H), 7.43 – 7.38 (m, 2H), 7.34 – 7.28 (m, 2H), 7.17 – 7.08 (m, 3H), 7.07 – 7.01 (m, 1H), 4.75 (ddq, *J* = 7.5, 7.0, 6.2 Hz, 1H), 4.08 (dd, *J* = 8.6, 7.6 Hz, 1H), 3.61 (dd, *J* = 8.6, 7.1 Hz, 1H), 1.50 (d, *J* = 6.2 Hz, 3H).

^{13}C NMR (101 MHz, $CDCl_3$): δ (ppm) = 149.0, 147.5, 139.9, 128.9, 128.6, 123.5, 122.9, 122.4, 118.8, 71.9, 52.9, 20.3.

IR (thin film, cm^{-1}): 2977, 2925, 2873, 1666, 1587, 1494, 1404, 1362, 1312, 1205, 1117, 1070, 976, 899, 846, 788, 751, 691, 658.

HRMS (ESI+): *m/z* for $C_{16}H_{17}N_2O$ $[M+H]^+$: calc.: 253.1335, found: 253.1334.

TLC: R_f = 0.46 (SiO_2, 20% EtOAc in hexane).

Compound 2j:

3-(4-Chlorophenyl)-5-methyl-*N*-(naphthalen-1-yl)oxazolidin-2-imine

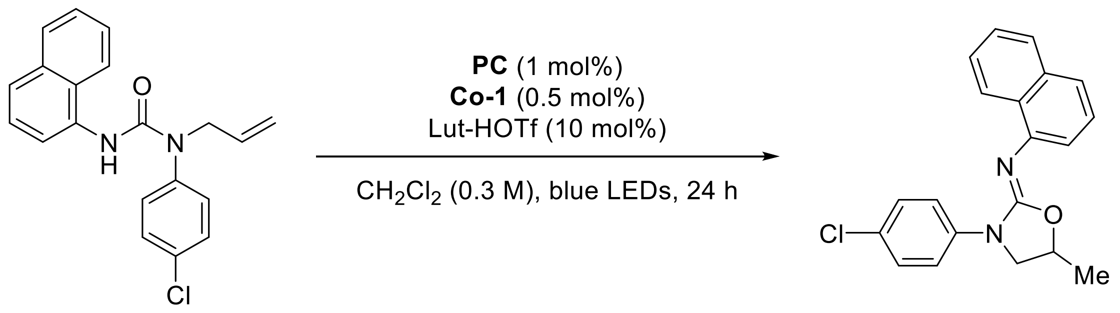

Isourea **2j** was prepared via GP1 from alkene **1j** (101 mg, 0.300 mmol, 1.00 equiv) in CH_2Cl_2. The crude product was purified via flash column chromatography (10-50% Et_2O in pentane) to give **2j** as an off-white solid.

Yield: 76.4 mg, 0.227 mmol, 76%

^{1}H NMR (400 MHz, $CDCl_3$): δ (ppm) = 8.22 – 8.16 (m, 1H), 7.90 – 7.79 (m, 3H), 7.58 – 7.52 (m, 1H), 7.50 – 7.34 (m, 5H), 7.20 (dd, *J* = 7.4, 1.2 Hz, 1H), 4.80 – 4.69 (m, 1H), 4.09 (dd, *J* = 8.6, 7.7 Hz, 1H), 3.62 (dd, *J* = 8.6, 7.0 Hz, 1H), 1.46 (d, *J* = 6.2 Hz, 3H).

^{13}C NMR (101 MHz, $CDCl_3$): δ (ppm) = 149.0, 143.8, 138.6, 134.5, 129.2, 128.9, 128.0, 127.9, 126.0, 125.8, 125.1, 124.3, 122.6, 119.9, 117.7, 72.0, 53.0, 20.3.

IR (thin film, cm^{-1}): 3051, 2978, 2929, 2881, 1666, 1593, 1572, 1493, 1423, 1388, 1362, 1309, 1215, 1125, 1094, 1071, 1040, 973, 907, 881, 824, 802, 773, 729, 694.

HRMS (ESI+): *m/z* for $C_{20}H_{18}ClN_2O$ $[M+H]^+$: calc.: 337.1102, found: 337.1096.

TLC: R_f = 0.47 (SiO_2, 50% CH_2Cl_2 in hexane).

Compound 2k:

***N*-(4-bromophenyl)-3,5-dimethyloxazolidin-2-imine**

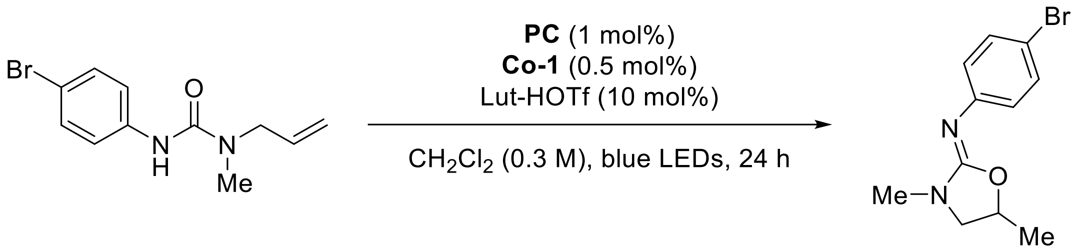

Isourea **2k** was prepared via GP1 from alkene **1k** (80.7 mg, 0.300 mmol, 1.00 equiv) in CH_2Cl_2. The crude product was purified via flash column chromatography (2-20% Et_2O in pentane) to give **2k** as a pale-yellow oil.

Yield: 75.4 mg, 0.280 mmol, 93%

^{1}H NMR (400 MHz, $CDCl_3$): δ (ppm) = 7.34 – 7.28 (m, 2H), 6.98 – 6.92 (m, 2H), 4.66 (tq, *J* = 7.4, 6.2 Hz, 1H), 3.61 (dd, *J* = 8.3, 7.6 Hz, 1H), 3.11 (dd, *J* = 8.3, 7.2 Hz, 1H), 2.97 (s, 3H), 1.43 (d, *J* = 6.2 Hz, 3H).

^{13}C NMR (101 MHz, $CDCl_3$): δ (ppm) = 154.1, 147.0, 131.5, 125.5, 114.7, 73.1, 55.0, 32.4, 20.2.

IR (thin film, cm^{-1}): 2930, 2850, 1667, 1581, 1493, 1403, 1362, 1334, 1269, 1223, 1069, 1006, 832, 786, 711.

HRMS (ESI+): *m/z* for $C_{11}H_{14}BrN_2O$ $[M+H]^+$: calc.: 269.0284, found: 269.0283.

TLC: R_f = 0.13 (SiO_2, 5% EtOAc in CH_2Cl_2).

Compound 2l:

5,5-Dimethyl-*N*,3-diphenyloxazolidin-2-imine

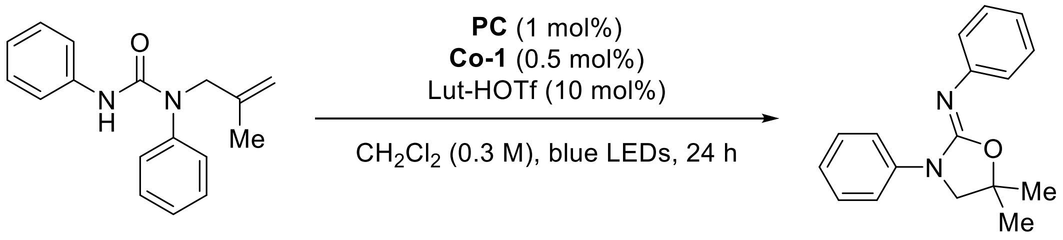

Isourea **2l** was prepared via GP1 from alkene **1l** (79.9 mg, 0.300 mmol, 1.00 equiv) in CH_2Cl_2. The crude product was purified via flash column chromatography (2-10% Et_2O in pentane) to give **2l** as a pale-yellow oil.

Yield: 70.6 mg, 0.265 mmol, 88%

^{1}H NMR (400 MHz, $CDCl_3$): δ (ppm) = 7.81 – 7.76 (m, 2H), 7.43 – 7.37 (m, 2H), 7.33 – 7.27 (m, 2H), 7.19 – 7.07 (m, 3H), 7.05 – 6.99 (m, 1H), 3.77 (s, 2H), 1.55 (s, 6H).

^{13}C NMR (101 MHz, $CDCl_3$): δ (ppm) = 148.9, 147.6, 140.2, 128.9, 128.5, 123.6, 122.8, 122.2, 118.8, 79.2, 58.1, 27.0.

IR (thin film, cm^{-1}): 3060, 2977, 2931, 2875, 1667, 1589, 1498, 1405, 1324, 1255, 1209, 1180, 1112, 1073, 1026, 980, 897, 751, 693, 658.

HRMS (ESI+): *m/z* for $C_{17}H_{19}N_2O$ $[M+H]^+$: calc.: 267.1492, found: 267.1486.

TLC: R_f = 0.53 (SiO_2, 20% EtOAc in hexane).

Compound 2m:

4-Methyl-*N*-(5-methyl-3-phenyloxazolidin-2-ylidene)benzenesulfonamide

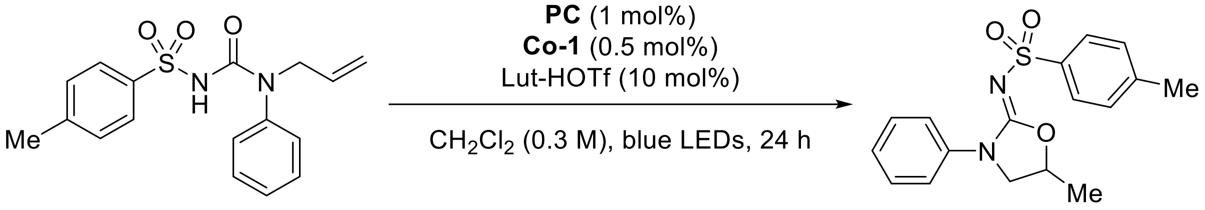

Isourea **2m** was prepared via GP1 from alkene **1m** (99.1 mg, 0.300 mmol, 1.00 equiv) in CH_2Cl_2. The crude material was purified via flash column chromatography (2-100% Et_2O in pentane) to give **2m** as a pale-yellow oil.

Yield: 55.6 mg, 0.168 mmol, 56%

^{1}H NMR (400 MHz, $CDCl_3$): δ (ppm) = 7.89 – 7.84 (m, 2H), 7.53 – 7.47 (m, 2H), 7.39 – 7.33 (m, 2H), 7.26 – 7.23 (m, 2H), 7.21 – 7.15 (m, 1H), 5.05 – 4.94 (m, 1H), 4.15 (dd, *J* = 9.1, 8.3 Hz, 1H), 3.67 (dd, *J* = 9.1, 7.2 Hz, 1H), 2.40 (s, 3H), 1.49 (d, *J* = 6.3 Hz, 3H).

^{13}C NMR (101 MHz, $CDCl_3$): δ (ppm) = 155.4, 142.5, 140.1, 137.0, 129.3, 129.1, 127.2, 125.8, 120.9, 75.2, 53.6, 21.6, 20.1.

IR (thin film, cm^{-1}): 3064, 2985, 1609, 1590, 1575, 1499, 1460, 1436, 1301, 1286, 1169, 1145, 1088, 1061, 1010, 865, 816, 759, 690, 669, 650.

HRMS (ESI+): *m/z* for $C_{17}H_{19}N_2O_3S$ $[M+H]^+$: calc.: 331.1111, found: 331.1106.

TLC: R_f = 0.16 (SiO_2, 50% EtOAc in hexane).

Compound 2n:

5-Methyl-3-phenyl-*N*-(*p*-tolyl)thiazolidin-2-imine

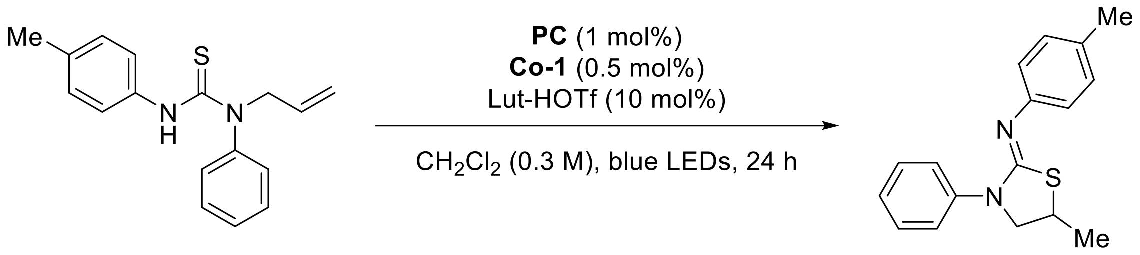

Isothiourea **2n** was prepared via GP1 from alkene **1n** (84.7 mg, 0.300 mmol, 1.00 equiv) in CH_2Cl_2. The crude product was purified via flash column chromatography (5-20% Et_2O in pentane) to give **2n** as an off-white solid.

Yield: 70.1 mg, 0.248 mmol, 83%

^{1}H NMR (400 MHz, $CDCl_3$): δ (ppm) = 7.61 – 7.53 (m, 2H), 7.42 – 7.33 (m, 2H), 7.17 – 7.05 (m, 3H), 6.90 – 6.82 (m, 2H), 4.22 – 4.11 (m, 1H), 3.82 – 3.69 (m, 2H), 2.31 (s, 3H), 1.47 (d, *J* = 6.4 Hz, 3H).

^{13}C NMR (101 MHz, $CDCl_3$): δ (ppm) = 156.9, 149.6, 141.6, 132.8, 129.5, 128.9, 124.3, 122.6, 121.7, 60.1, 37.5, 21.1, 20.2.

IR (thin film, cm^{-1}): 3022, 2921, 2863, 1623, 1605, 1589, 1497, 1466, 1454, 1386, 1327, 1295, 1264, 1177, 1159, 1106, 1089, 1012, 960, 868, 837, 776, 754, 691, 628.

HRMS (ESI+): *m/z* for $C_{17}H_{19}N_2S$ $[M+H]^+$: calc.: 283.1263, found: 283.1261.

TLC: R_f = 0.58 (SiO_2, 20% EtOAc in hexane).

Compound 2o:

4-Methyl-1,3-dioxan-2-one

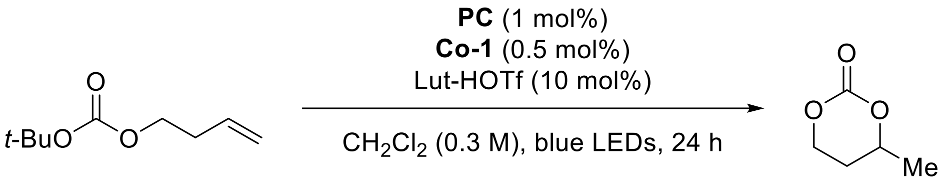

Carbonate **2o** was prepared via GP1 from alkene **1o** (51.7 mg, 0.300 mmol, 1.00 equiv) in CH_2Cl_2. The crude product was purified via flash column chromatography (20-100% Et_2O in pentane) to give **2o** as a colorless oil.

Yield: 27.0 mg, 0.233 mmol, 78%

^{1}H NMR (400 MHz, $CDCl_3$): δ (ppm) = 4.67 – 4.57 (m, 1H), 4.48 – 4.33 (m, 2H), 2.08 (dtd, *J* = 14.4, 3.6, 3.0 Hz, 1H), 1.92 (dddd, *J* = 14.4, 11.1, 10.2, 5.3 Hz, 1H), 1.44 (d, *J* = 6.3 Hz, 3H).

^{13}C NMR (101 MHz, $CDCl_3$): δ (ppm) = 149.0, 75.8, 67.0, 28.8, 21.3.

IR (thin film, cm^{-1}): 2984 2922, 1739, 1668, 1590, 1484, 1408, 1351, 1324, 1241, 1199, 1155, 1113, 768, 668.

HRMS (ESI+): *m/z* for $C_5H_8NaO_3$ $[M+Na]^+$: calc.: 139.0366, found: 139.0366.

TLC: R_f = 0.43 (SiO_2, EtOAc).

Compound 2p:

4,4-Dimethyl-1,3-dioxan-2-one

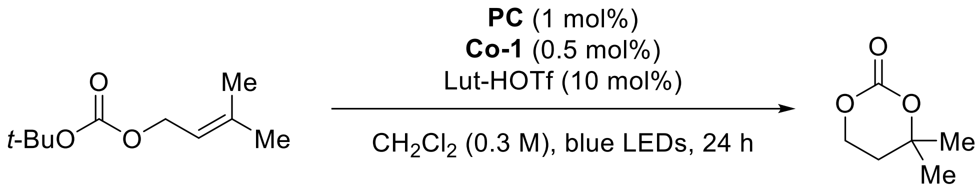

Carbonate **2p** was prepared via GP1 from alkene **1p** (55.9 mg, 0.300 mmol, 1.00 equiv) in CH_2Cl_2. The crude product was purified via flash column chromatography (20-100% Et_2O in pentane) to give **2p** as a white solid.

Yield: 17.7 mg, 0.136 mmol, 45%

^{1}H NMR (400 MHz, $CDCl_3$): δ (ppm) = 4.47 – 4.39 (m, 2H), 2.04 – 1.97 (m, 2H), 1.48 (s, 6H).

^{13}C NMR (101 MHz, $CDCl_3$): δ (ppm) = 149.2, 81.3, 64.8, 32.9, 28.0.

IR (thin film, cm^{-1}): 2985, 2932, 1721, 1412, 1290, 1275, 1218, 1197, 1131, 1107, 1056, 1023, 771, 666.

HRMS (ESI+): *m/z* for $C_6H_{10}NaO_3$ $[M+Na]^+$: calc.: 153.0522, found: 153.0521.

TLC: R_f = 0.17 (SiO_2, 50% EtOAc in hexane).

Compound 2q:

4,4-Diphenyl-1,3-dioxan-2-one

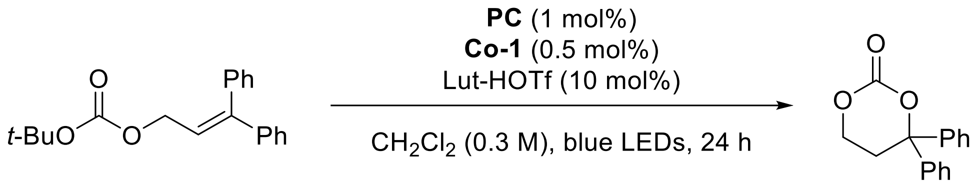

Carbonate **2q** was prepared via GP1 from alkene **1q** (93.1 mg, 0.300 mmol, 1.00 equiv) in CH_2Cl_2. The crude product was purified via flash column chromatography (10-50% Et_2O in pentane) to give **2q** as a white solid.

Yield: 63.9 mg, 0.251 mmol, 84%

^{1}H NMR (400 MHz, $CDCl_3$): δ (ppm) = 7.43 – 7.29 (m, 10H), 4.36 – 4.29 (m, 2H), 2.85 – 2.78 (m, 2H).

^{13}C NMR (101 MHz, $CDCl_3$): δ (ppm) = 148.5, 142.0, 129.0, 128.5, 125.5, 87.0, 65.3, 32.4.

IR (thin film, cm^{-1}): 3061, 2922, 2854, 1740, 1494, 1475, 1449, 1404, 1265, 1216, 1185, 1109, 1060, 1032, 1003, 764, 752, 697, 621, 607.

HRMS (ESI+): *m/z* for $C_{16}H_{14}NaO_3$ $[M+Na]^+$: calc.: 277.0835, found: 277.0834.

TLC: R_f = 0.13 (SiO_2, 20% EtOAc in hexane).

Compound 2r:

1-Benzyl-4-methyl-3-tosylimidazolidin-2-one

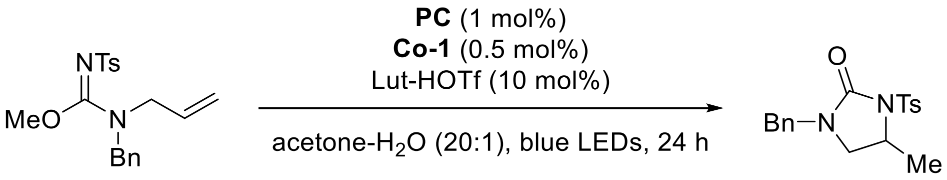

Urea **2r** was prepared via GP1 from alkene **1r** (108 mg, 0.300 mmol, 1.00 equiv) in acetone-H_2O (20:1). The crude product was purified via flash column chromatography (2-50% Et_2O in pentane) to give **2r** as a pale-yellow oil.

Yield: 56.7 mg, 0.165 mmol, 55%

^{1}H NMR (400 MHz, $CDCl_3$): δ (ppm) 7.99 – 7.94 (m, 2H), 7.36 – 7.25 (m, 5H), 7.16 – 7.11 (m, 2H), 4.39 – 4.23 (m, 3H), 3.40 (t, *J* = 8.9 Hz, 1H), 2.79 (dd, *J* = 9.0, 4.1 Hz, 1H), 2.45 (s, 3H), 1.45 (d, *J* = 6.2 Hz, 3H).

^{13}C NMR (101 MHz, $CDCl_3$): δ (ppm) = 154.0, 144.6, 136.6, 135.6, 129.7, 128.9, 128.3, 128.2, 128.1, 50.6, 49.3, 47.7, 22.0, 21.8.

IR (thin film, cm^{-1}): 2924, 2863, 1726, 1597, 1494, 1442, 1358, 1166, 1090, 816, 752, 726, 703, 664.

HRMS (ESI+): *m/z* for $C_{18}H_{20}N_2NaO_3S$ $[M+Na]^+$: calc.: 367.1087, found: 367.1083.

TLC: R_f = 0.17 (SiO_2, 20% EtOAc in hexane).

4. Preparation of Starting Materials

Starting materials were prepared according to literature-known procedures yielding the desired isoureas,[3] ureas,[3-4] thioureas,[5] guanidines,[6] and carbonates.[7] All three catalysts were prepared according to literature procedures.[8]

Compound 1j:

1-Allyl-1-(4-chlorophenyl)-3-(naphthalen-1-yl)urea

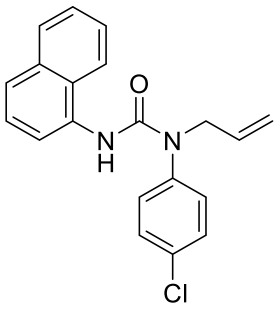

To a stirred solution of 1-isocyanatonaphthalene (510 mg, 3.0 mmol, 1.0 equiv.) in 10 mL anhydrous CH_2Cl_2 at 0 °C was added triethylamine (840 µL, 6.0 mmol, 2.0 equiv.) followed by *N*-allyl-4-chloroaniline (550 mg, 3.3 mmol, 1.1 equiv). The reaction was allowed to warm to room temperature and stirred for 4 h. The reaction mixture was then diluted with CH_2Cl_2, washed with water, and concentrated under reduced pressure. The residue was purified via flash column chromatography (5-20% Et_2O in pentane) to give **1j** as a white solid.

Yield: 735 mg, 2.18 mmol, 73%

^{1}H NMR (400 MHz, $CDCl_3$): δ (ppm) = 7.93 (dt, *J* = 7.5, 0.9 Hz, 1H), 7.84 – 7.79 (m, 1H), 7.61 (dt, *J* = 8.2, 1.1 Hz, 1H), 7.54 – 7.49 (m, 2H), 7.48 – 7.37 (m, 5H), 7.35 – 7.30 (m, 1H), 6.55 (s, 1H), 5.99 (ddt, *J* = 16.7, 10.6, 6.2 Hz, 1H), 5.22 – 5.12 (m, 2H), 4.39 (dt, *J* = 6.3, 1.3 Hz, 2H).

^{13}C NMR (101 MHz, $CDCl_3$): δ (ppm) = 154.4, 140.2, 134.3, 134.2, 134.0, 133.4, 130.7, 130.2, 128.9, 127.1, 126.2, 126.0, 125.8, 124.7, 120.2, 119.7, 118.1, 52.7.

IR (thin film, cm^{-1}): 3061, 2918, 1676, 1528, 1491, 1438, 1406, 1358, 1260, 1358, 1260, 1091, 1016, 792, 772.

HRMS (ESI+): *m/z* for $C_{20}H_{18}ClN_2O$ $[M+H]^+$: calc.: 337.1102, found: 337.1094.

TLC: R_f = 0.37 (SiO_2, 20% EtOAc in hexane).

5. ^{1}H and ^{13}C NMR Spectra

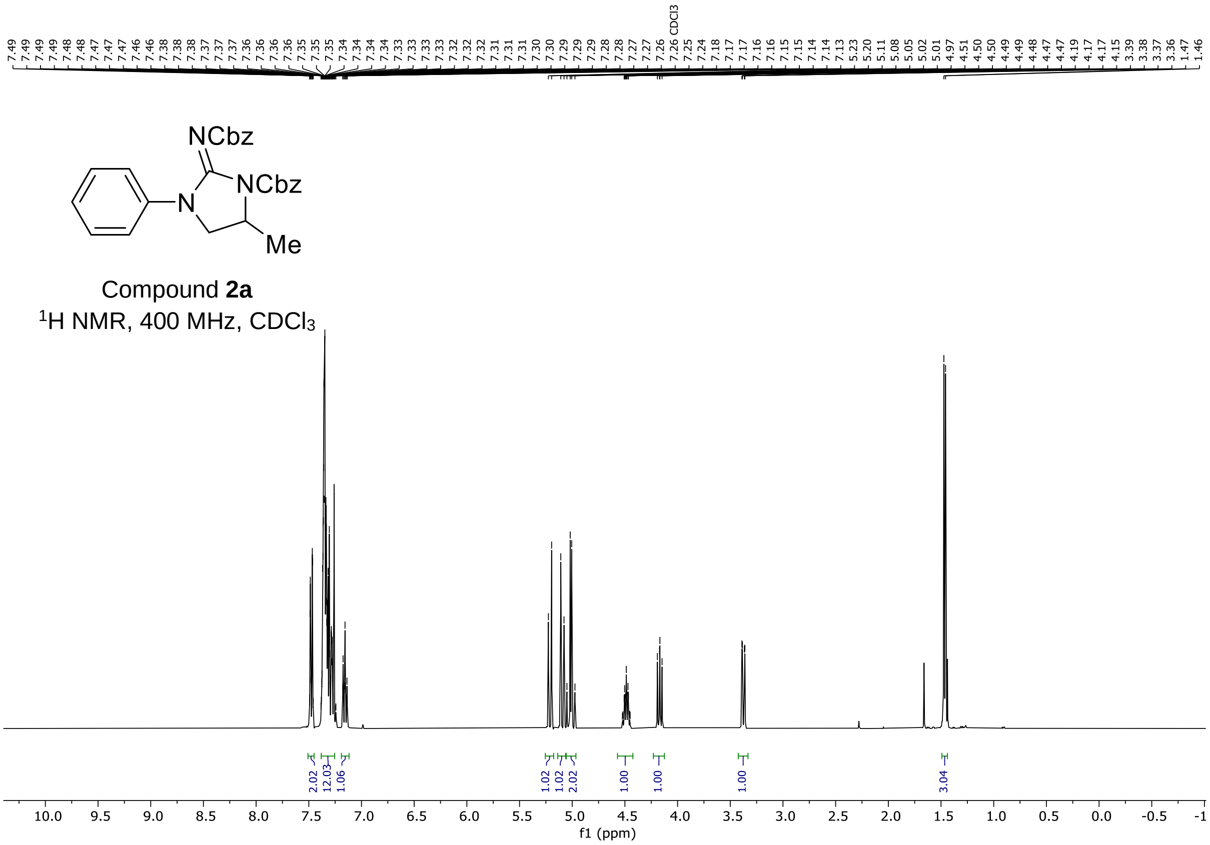

Compound **2a**
^{1}H NMR, 400 MHz, $CDCl_3$

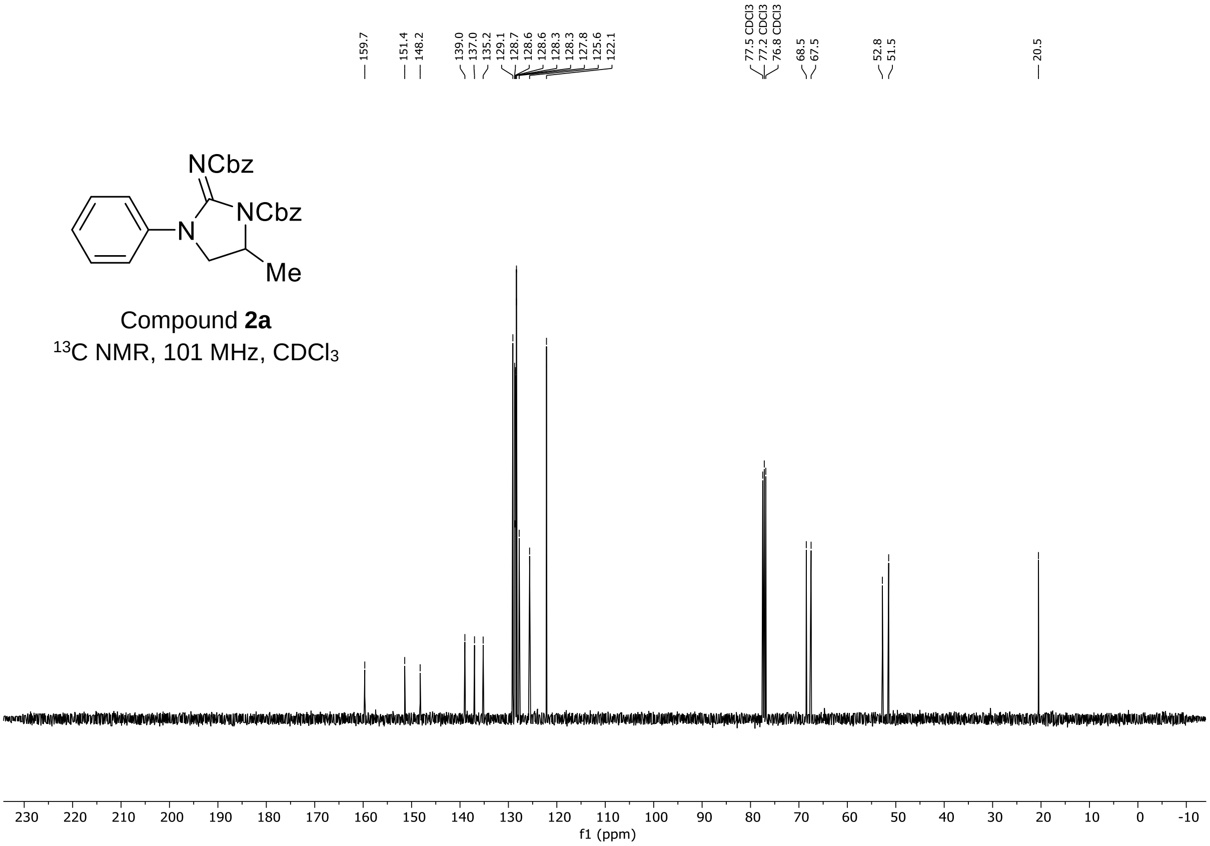

Compound **2a**
^{13}C NMR, 101 MHz, $CDCl_3$

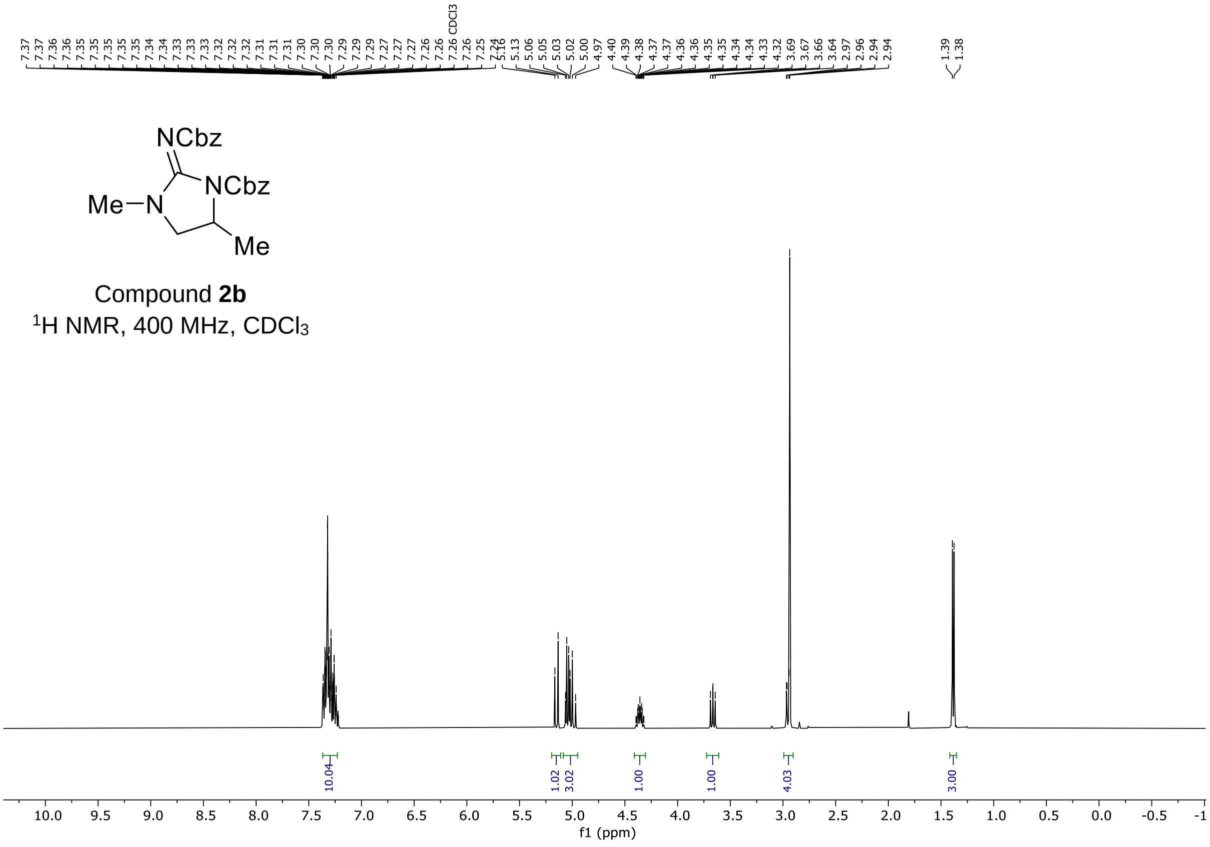
NCbz
NCbz
Me−N
Me
Compound **2b**
^{1}H NMR, 400 MHz, $CDCl_3$
f1 (ppm)

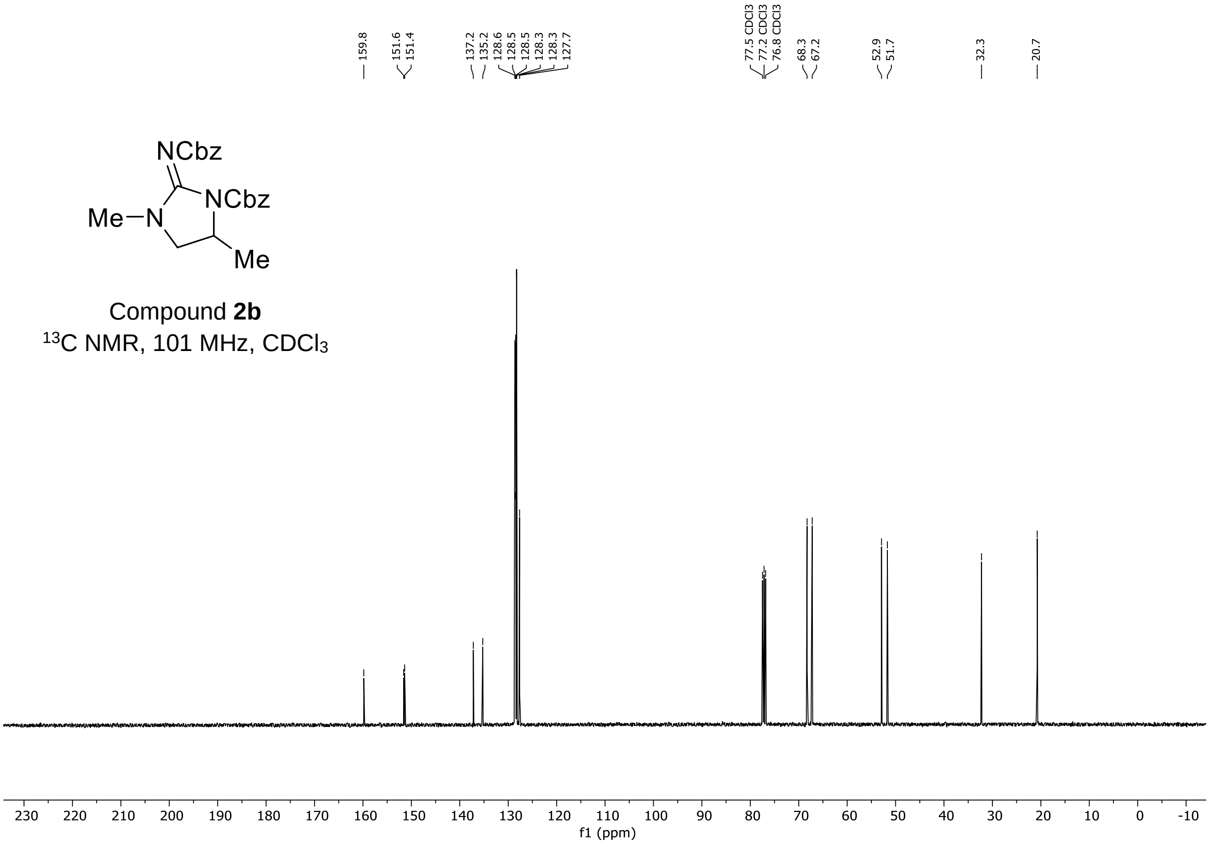
NCbz
NCbz
Me−N
Me
Compound **2b**
^{13}C NMR, 101 MHz, $CDCl_3$
f1 (ppm)

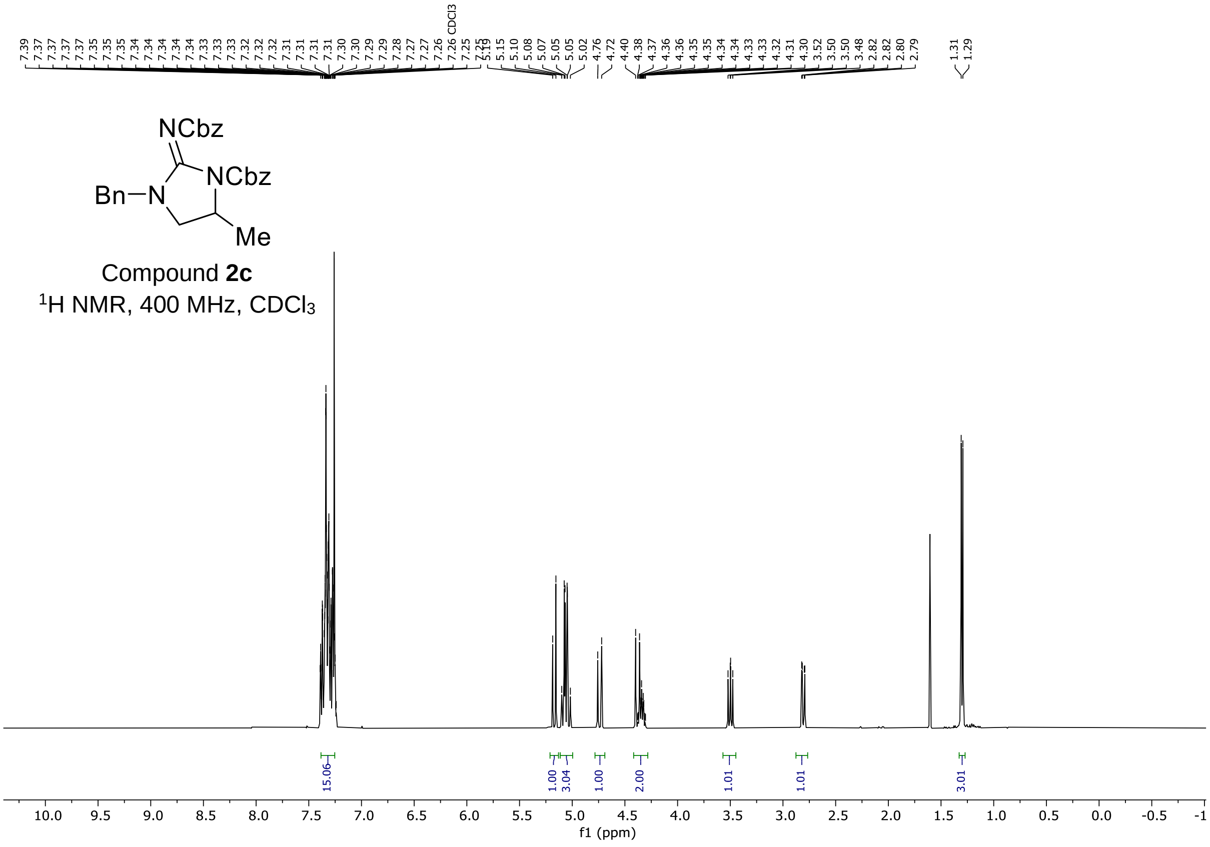
NCbz
Bn—N
NCbz
Me
Compound **2c**
^{1}H NMR, 400 MHz, $CDCl_3$
f1 (ppm)

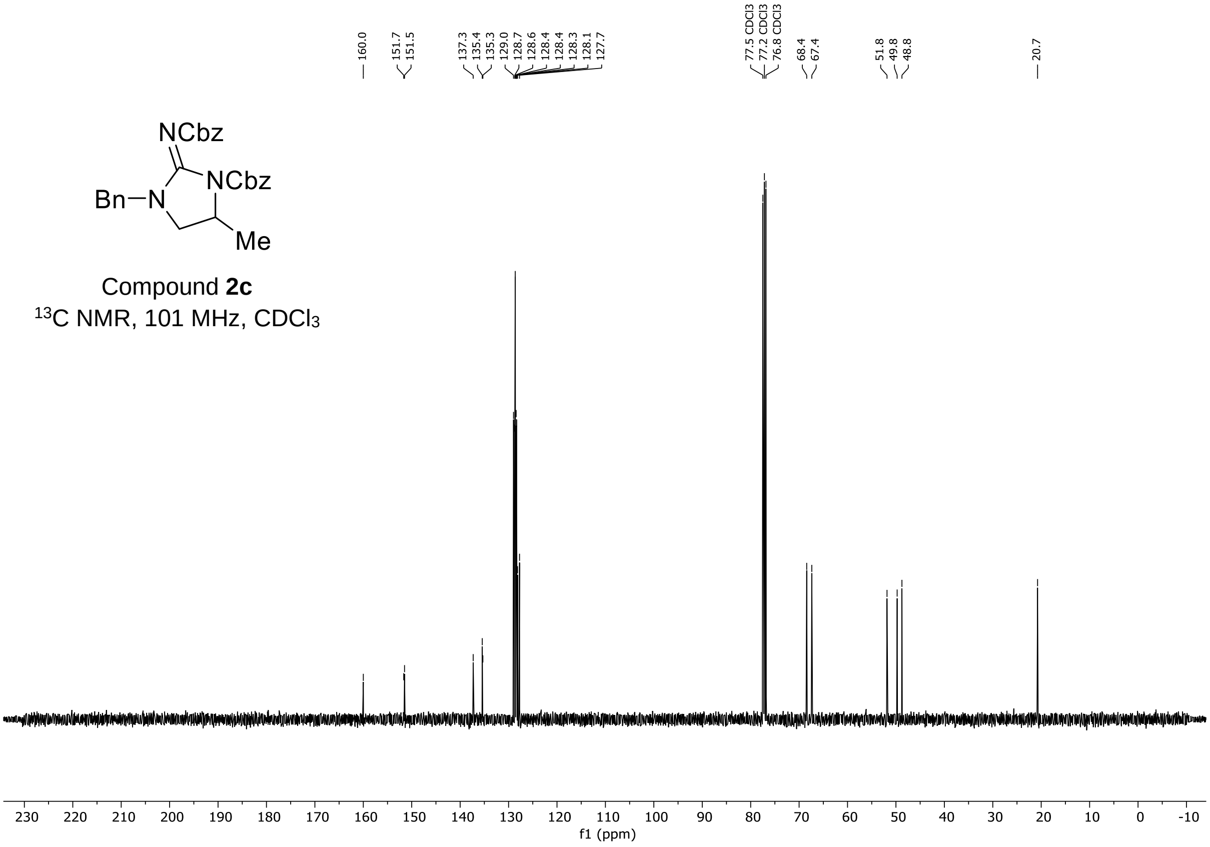
NCbz
Bn—N
NCbz
Me
Compound **2c**
^{13}C NMR, 101 MHz, $CDCl_3$
f1 (ppm)

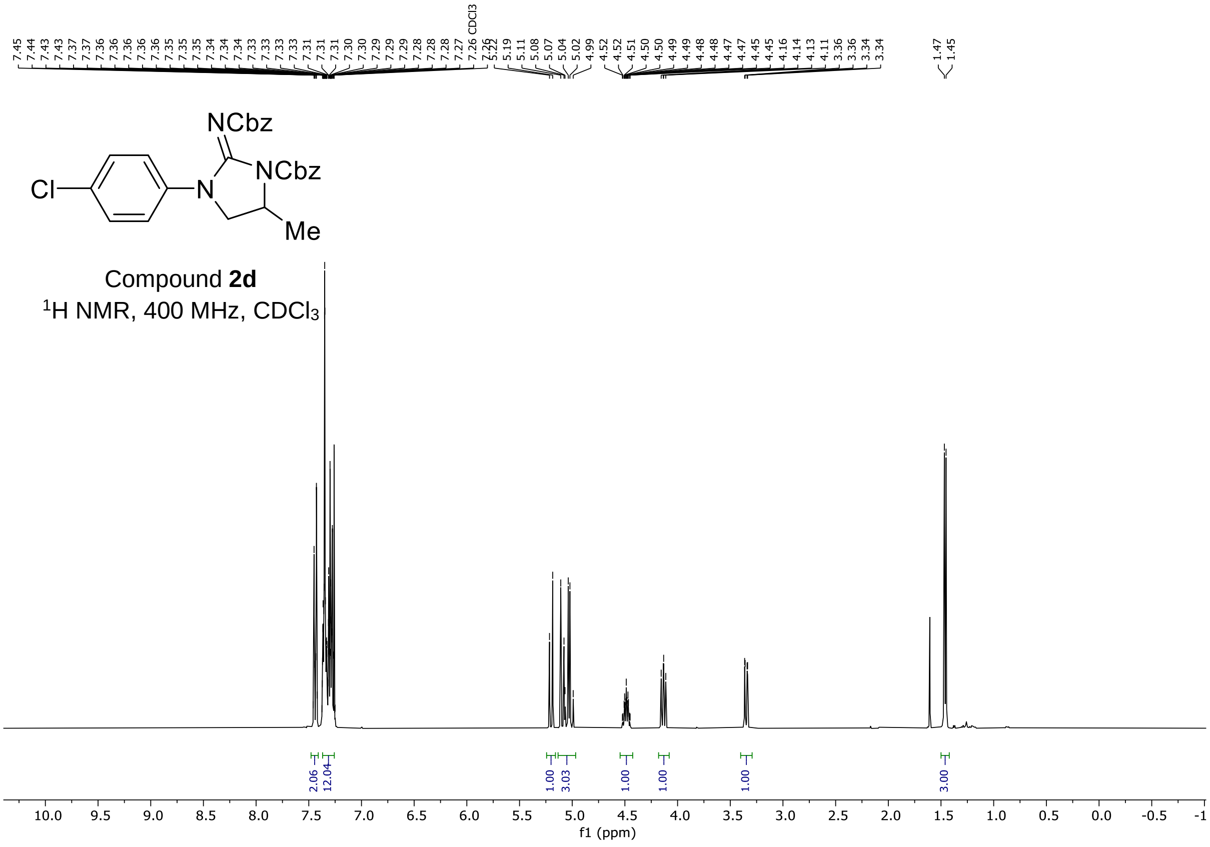
7.45 7.44 7.43 7.43 7.37 7.37 7.36 7.36 7.36 7.36 7.35 7.35 7.35 7.34 7.34 7.34 7.33 7.33 7.33 7.33 7.31 7.31 7.31 7.30 7.30 7.29 7.29 7.29 7.28 7.28 7.28 7.27 7.26 CDCl3
[illegible] 5.22 5.19 5.11 5.08 5.07 5.04 5.02 4.99 4.52 4.52 4.51 4.50 4.50 4.49 4.49 4.48 4.48 4.47 4.47 4.45 4.45 4.16 4.14 4.13 4.11 3.36 3.36 3.34 3.34
1.47 1.45
NCbz
Cl
N
NCbz
Me
Compound **2d**
^{1}H NMR, 400 MHz, $CDCl_3$
2.06 12.04 1.00 3.03 1.00 1.00 1.00 3.00
f1 (ppm)

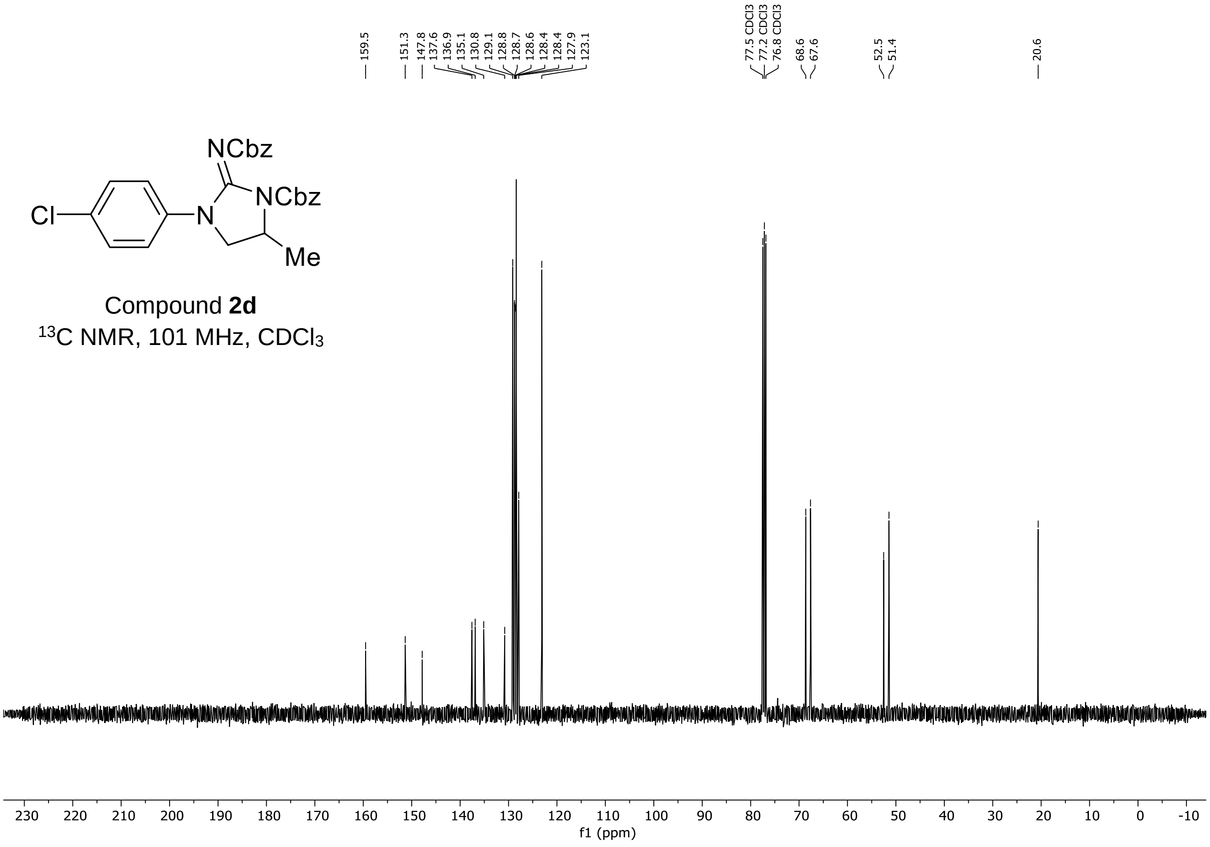
159.5 151.3 147.8 137.6 136.9 135.1 130.8 129.1 128.8 128.7 128.6 128.4 128.4 127.9 123.1
77.5 CDCl3 77.2 CDCl3 76.8 CDCl3 68.6 67.6 52.5 51.4 20.6
NCbz
Cl
N
NCbz
Me
Compound **2d**
^{13}C NMR, 101 MHz, $CDCl_3$
f1 (ppm)

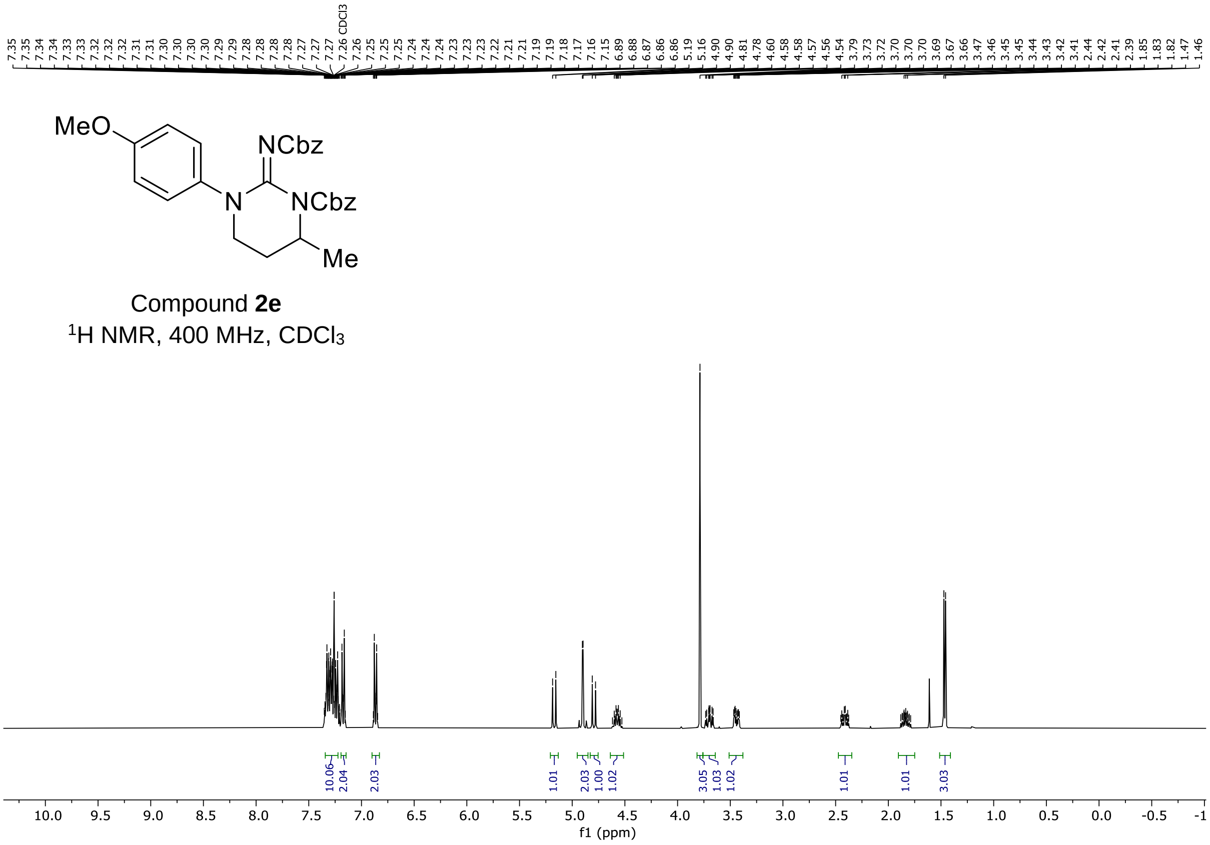

Compound **2e**
^{1}H NMR, 400 MHz, $CDCl_3$

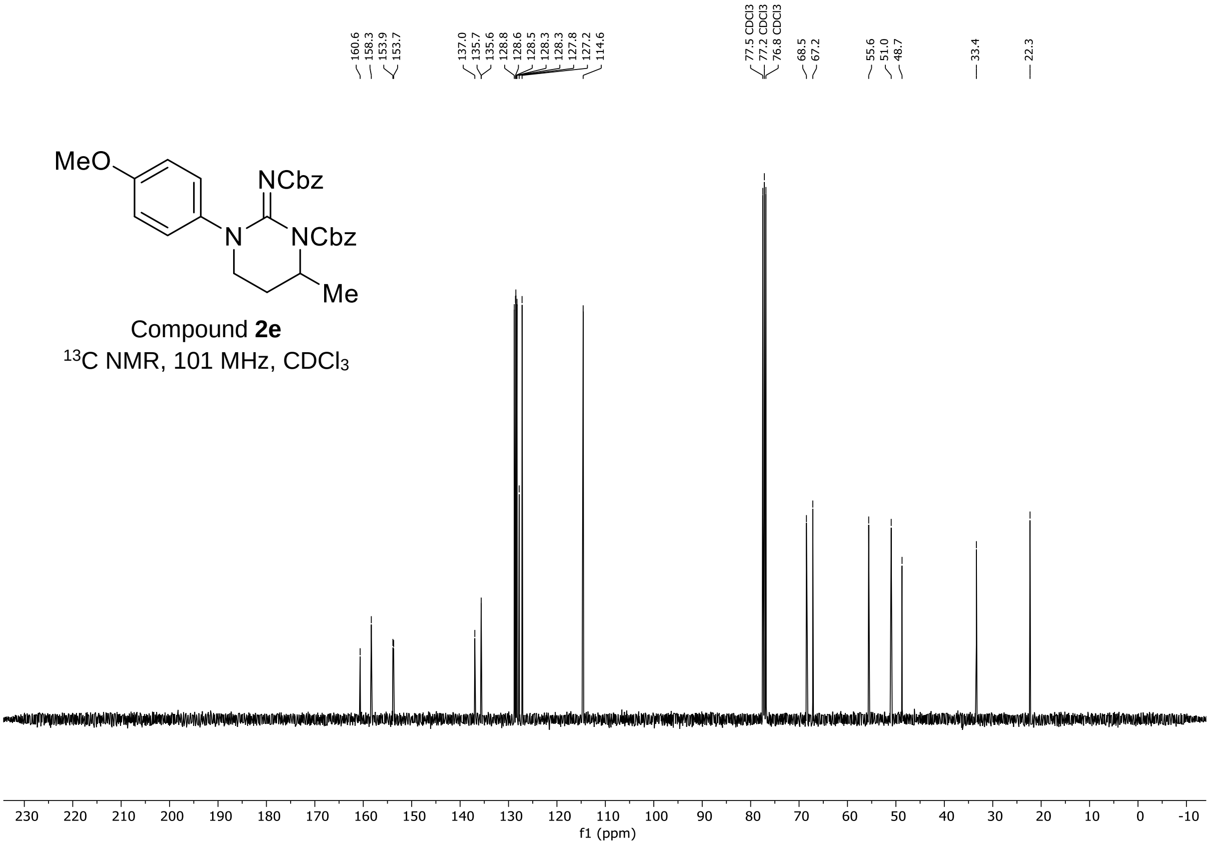

Compound **2e**
^{13}C NMR, 101 MHz, $CDCl_3$

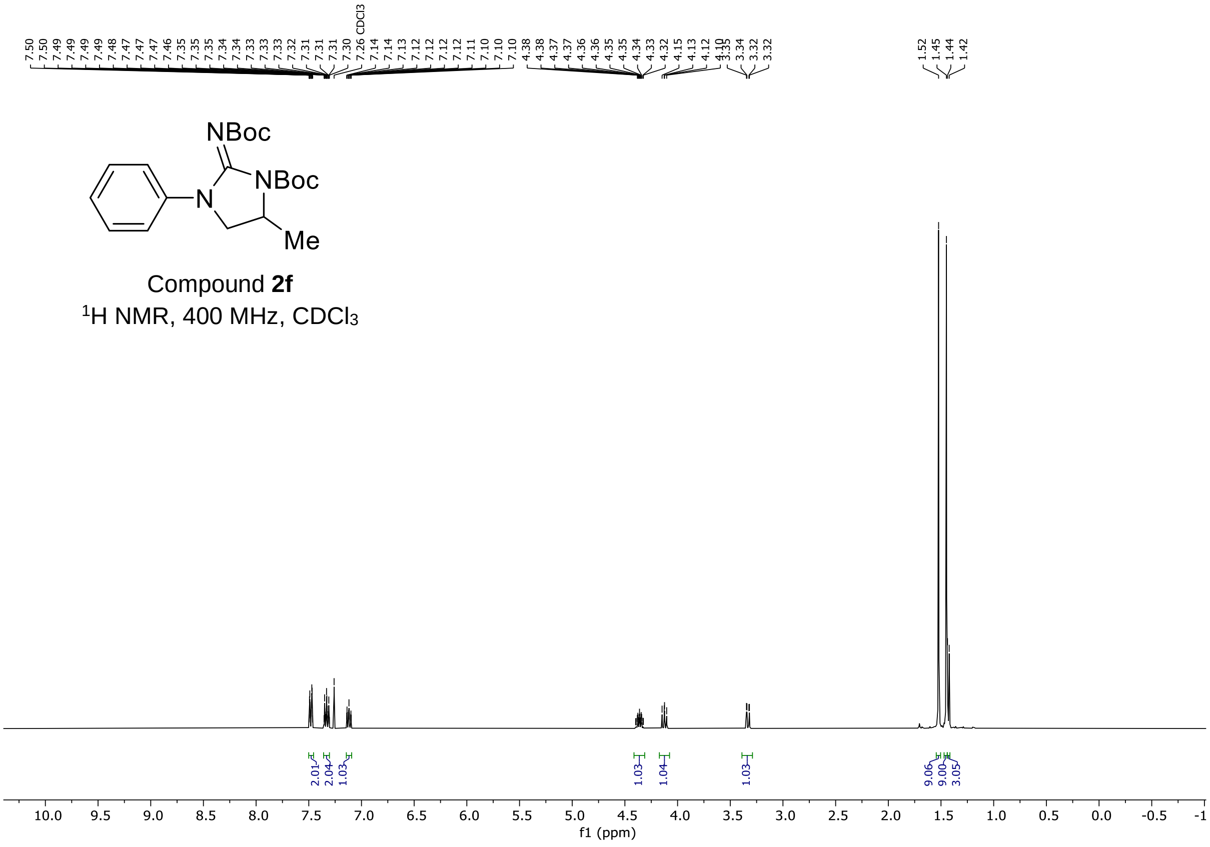
NBoc
NBoc
N
Me
Compound **2f**
^{1}H NMR, 400 MHz, $CDCl_3$
f1 (ppm)

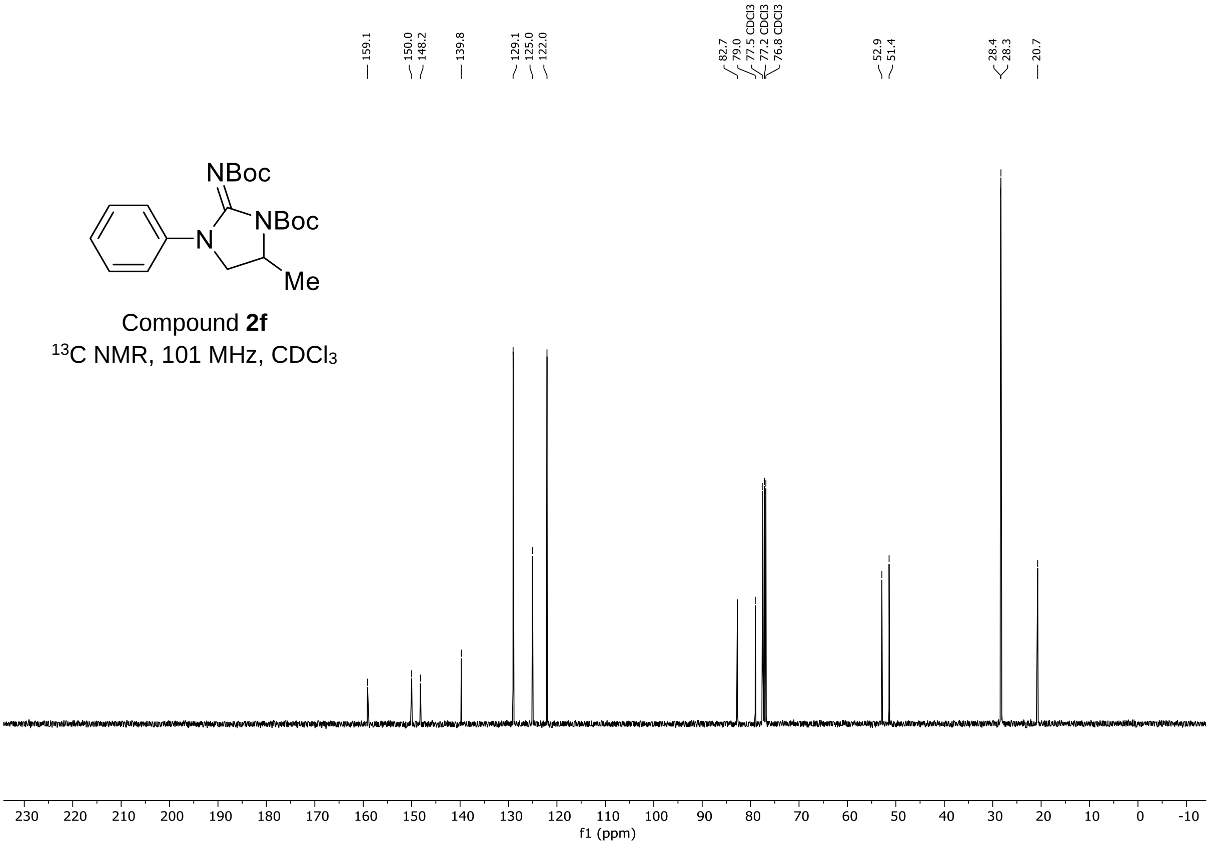
NBoc
NBoc
N
Me
Compound **2f**
^{13}C NMR, 101 MHz, $CDCl_3$
159.1
150.0
148.2
139.8
129.1
125.0
122.0
82.7
79.0
77.5 CDCl3
77.2 CDCl3
76.8 CDCl3
52.9
51.4
28.4
28.3
20.7
f1 (ppm)

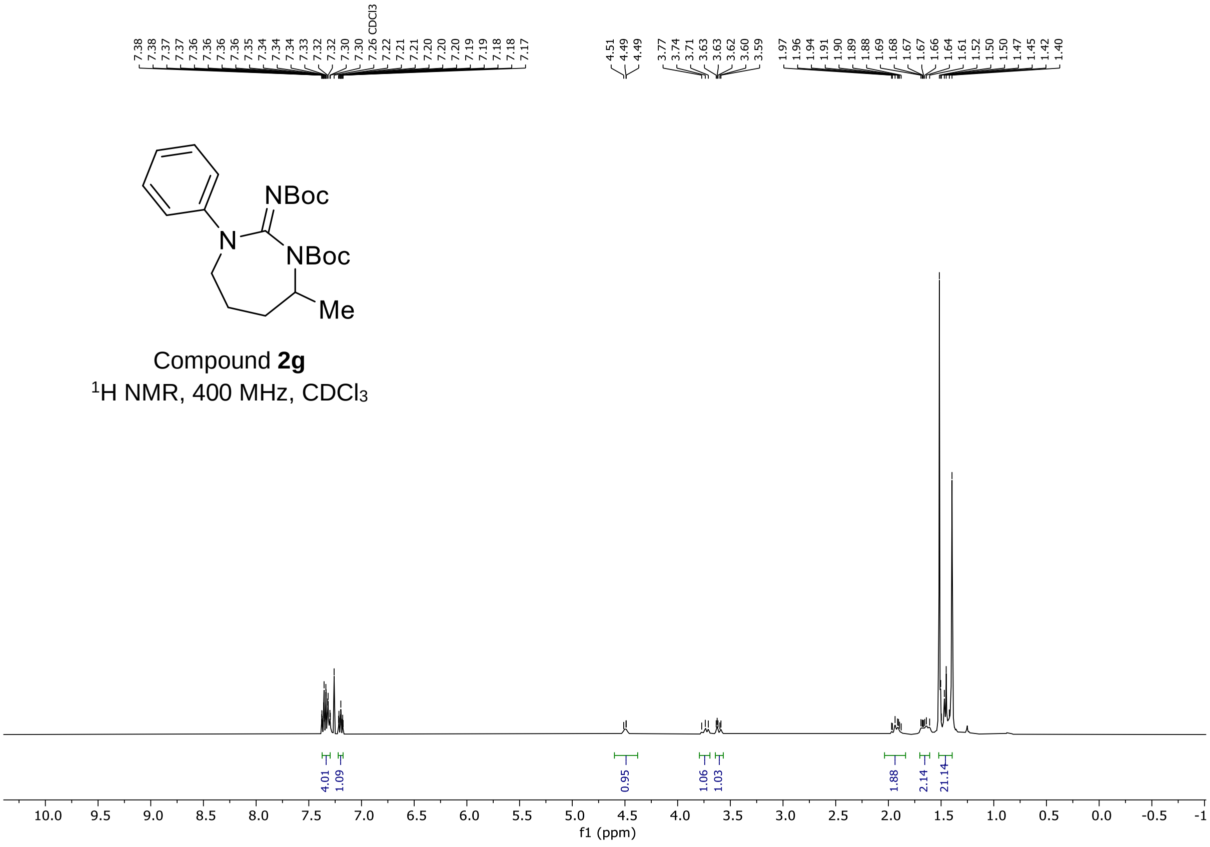

Compound **2g**
^{1}H NMR, 400 MHz, $CDCl_3$

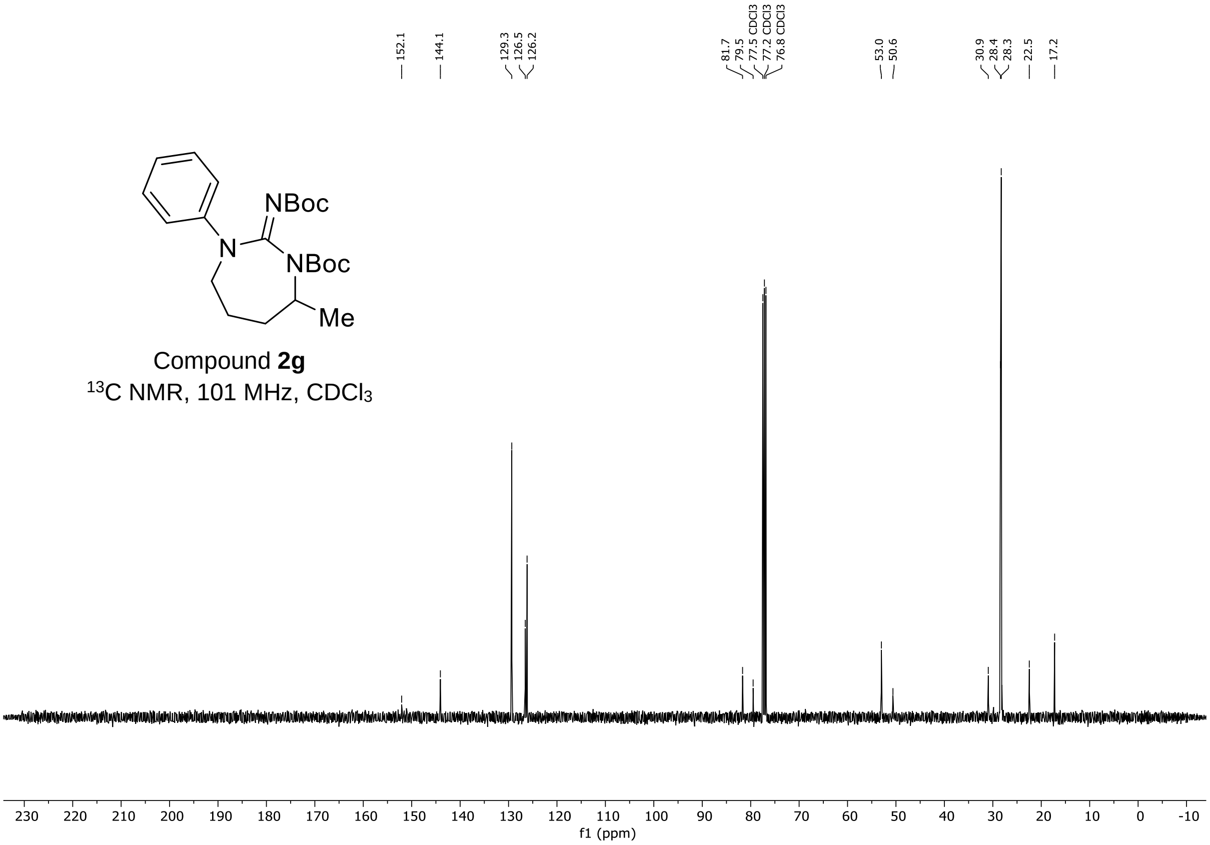

Compound **2g**
^{13}C NMR, 101 MHz, $CDCl_3$

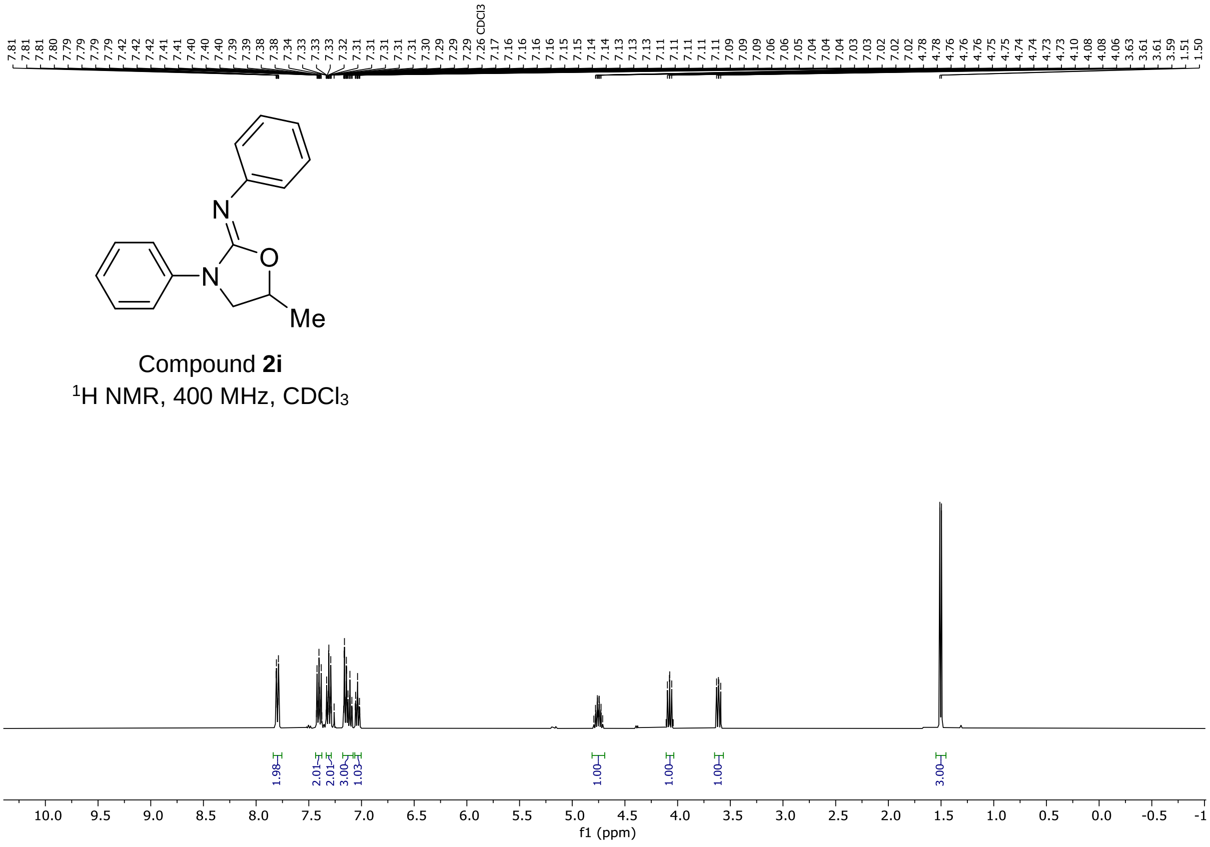

Compound **2i**
^{1}H NMR, 400 MHz, $CDCl_3$

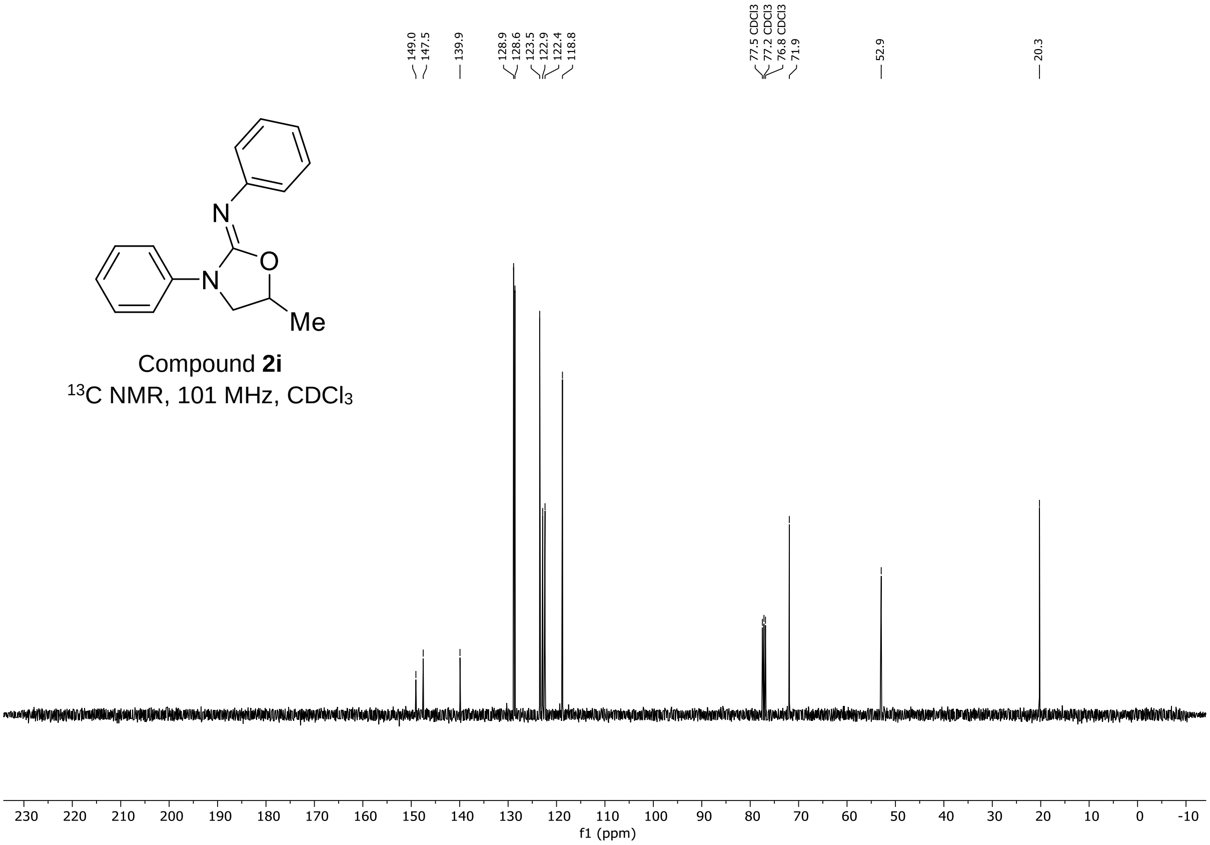

Compound **2i**
^{13}C NMR, 101 MHz, $CDCl_3$

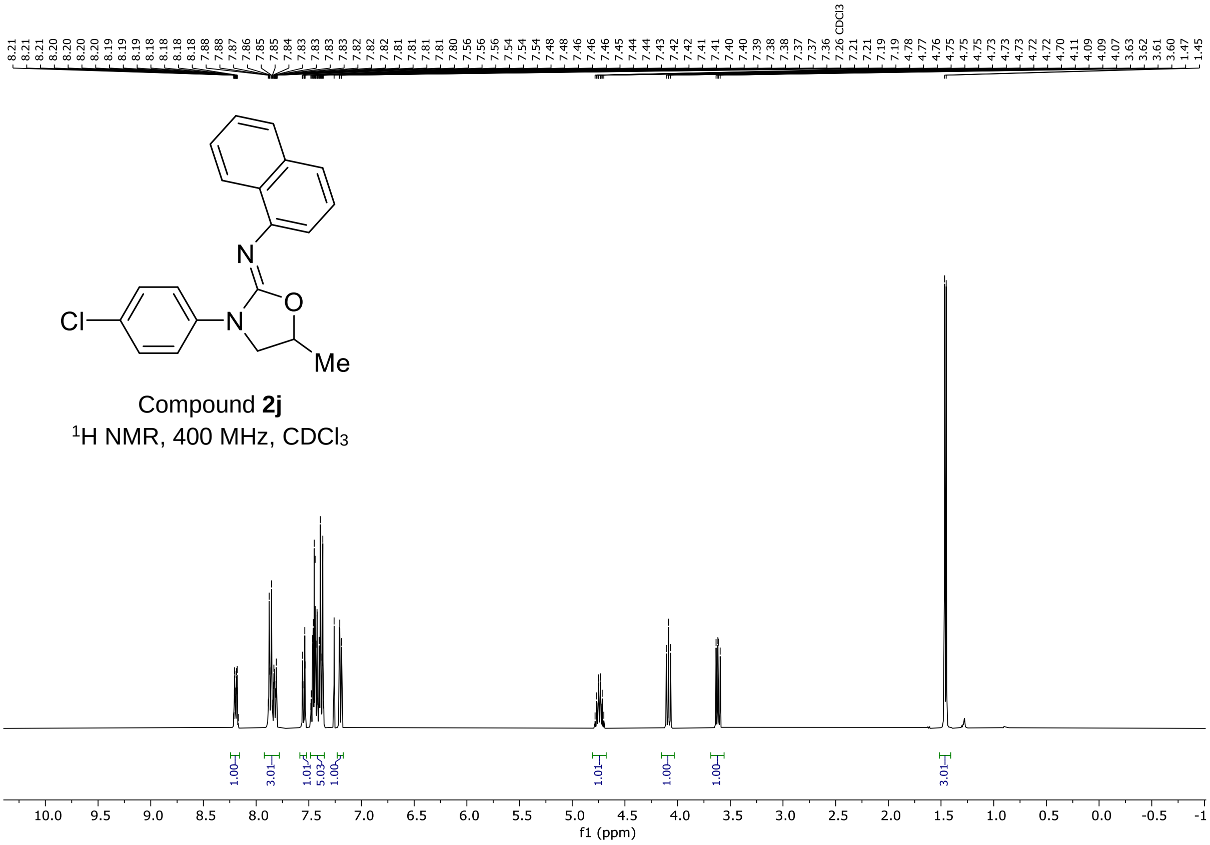
8.21 8.21 8.21 8.20 8.20 8.20 8.19 8.19 8.19 8.18 8.18 8.18 8.18 7.88 7.87 7.86 7.85 7.85 7.84 7.83 7.83 7.83 7.83 7.82 7.82 7.81 7.81 7.81 7.81 7.80 7.56 7.56 7.56 7.54 7.54 7.54 7.48 7.48 7.46 7.46 7.45 7.44 7.44 7.43 7.42 7.42 7.41 7.41 7.40 7.40 7.39 7.38 7.38 7.37 7.37 7.36 7.26 CDCl3 7.21 7.21 7.19 7.19 4.78 4.77 4.76 4.75 4.75 4.73 4.73 4.73 4.72 4.72 4.70 4.11 4.09 4.07 3.63 3.62 3.61 3.60 1.47 1.45
N
O
Cl
N
Me
Compound 2j
1H NMR, 400 MHz, CDCl3
1.00 3.01 1.01 5.03 1.00 1.01 1.00 1.00 3.01
10.0 9.5 9.0 8.5 8.0 7.5 7.0 6.5 6.0 5.5 5.0 4.5 4.0 3.5 3.0 2.5 2.0 1.5 1.0 0.5 0.0 -0.5 -1
f1 (ppm)

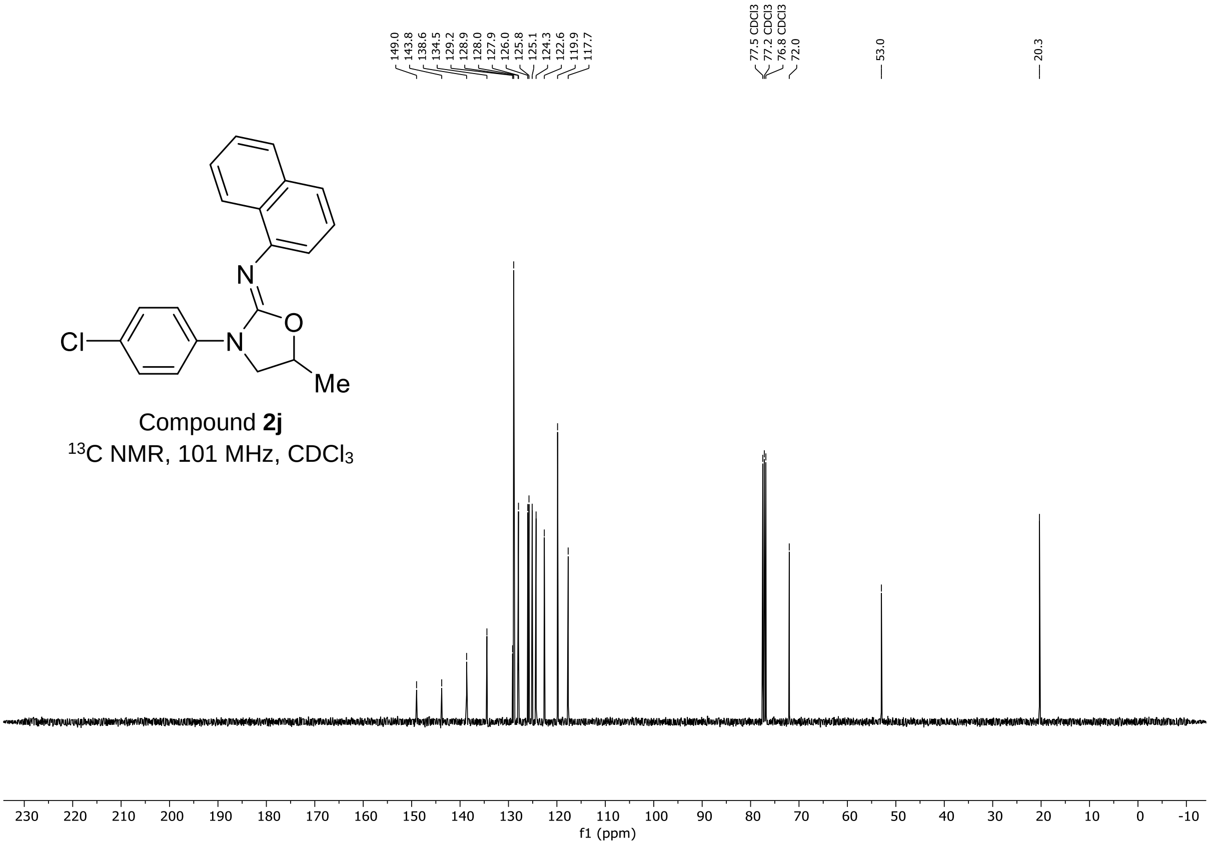
149.0 143.8 138.6 134.5 129.2 128.9 128.0 127.9 126.0 125.8 125.1 124.3 122.6 119.9 117.7
77.5 CDCl3 77.2 CDCl3 76.8 CDCl3 72.0
53.0
20.3
N
O
Cl
N
Me
Compound 2j
13C NMR, 101 MHz, CDCl3
230 220 210 200 190 180 170 160 150 140 130 120 110 100 90 80 70 60 50 40 30 20 10 0 -10
f1 (ppm)

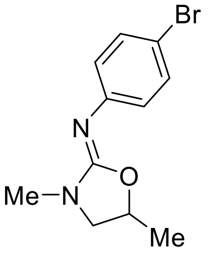

Compound **2k**

^{1}H NMR, 400 MHz, $CDCl_3$

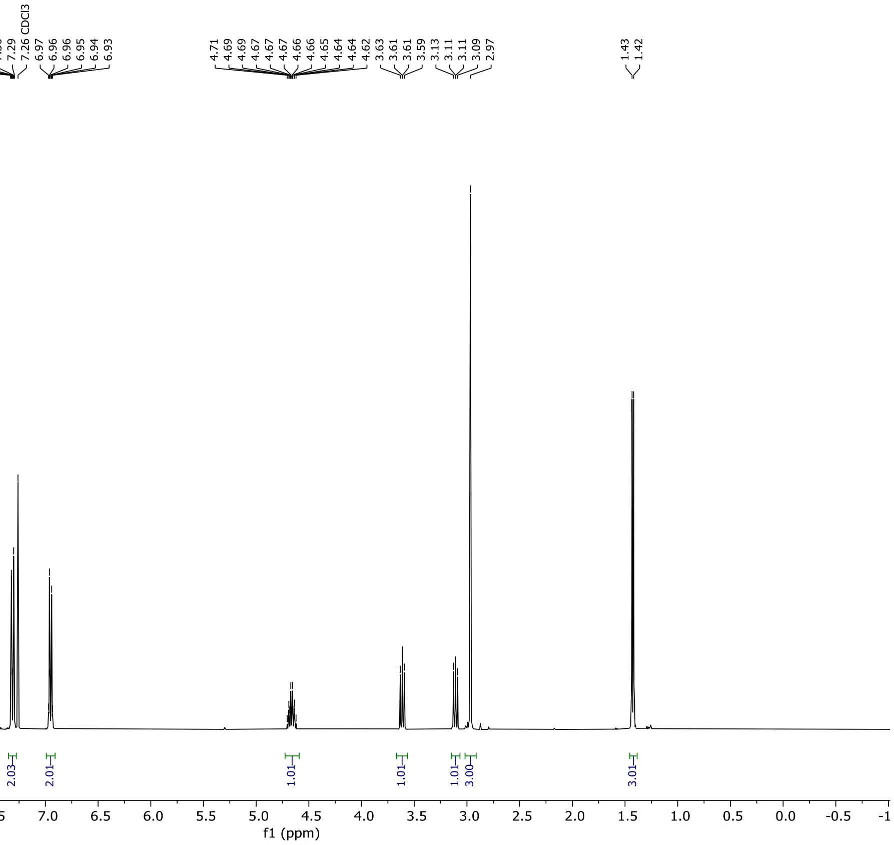

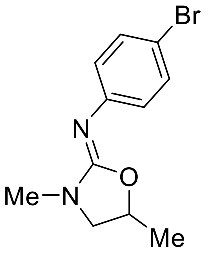

Compound **2k**

^{13}C NMR, 101 MHz, $CDCl_3$

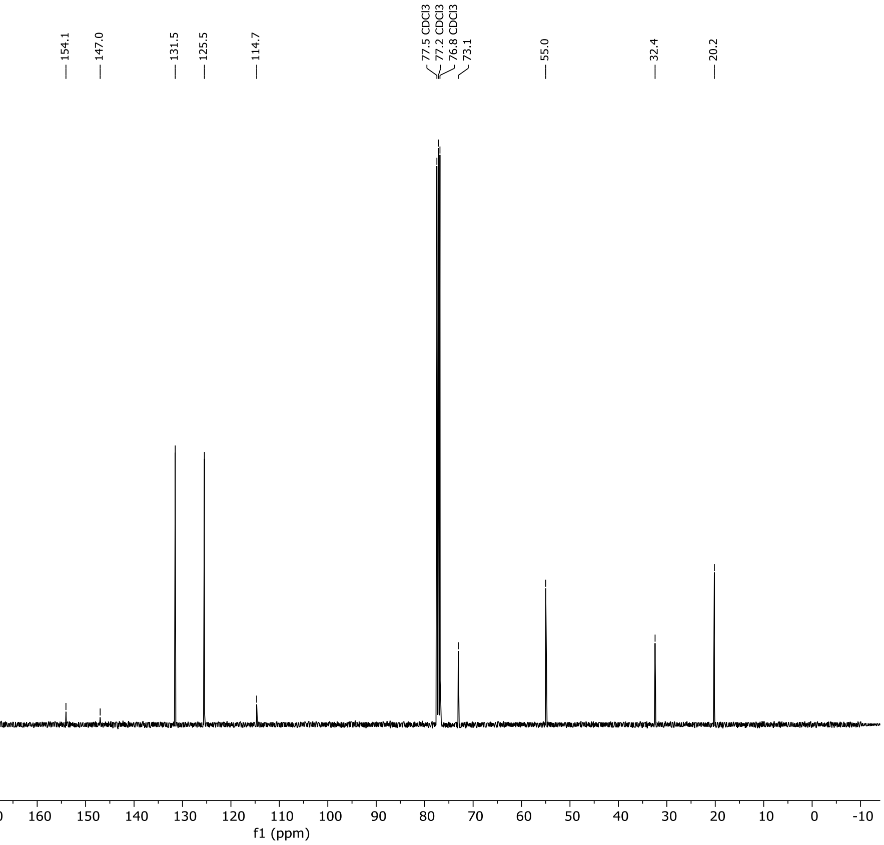

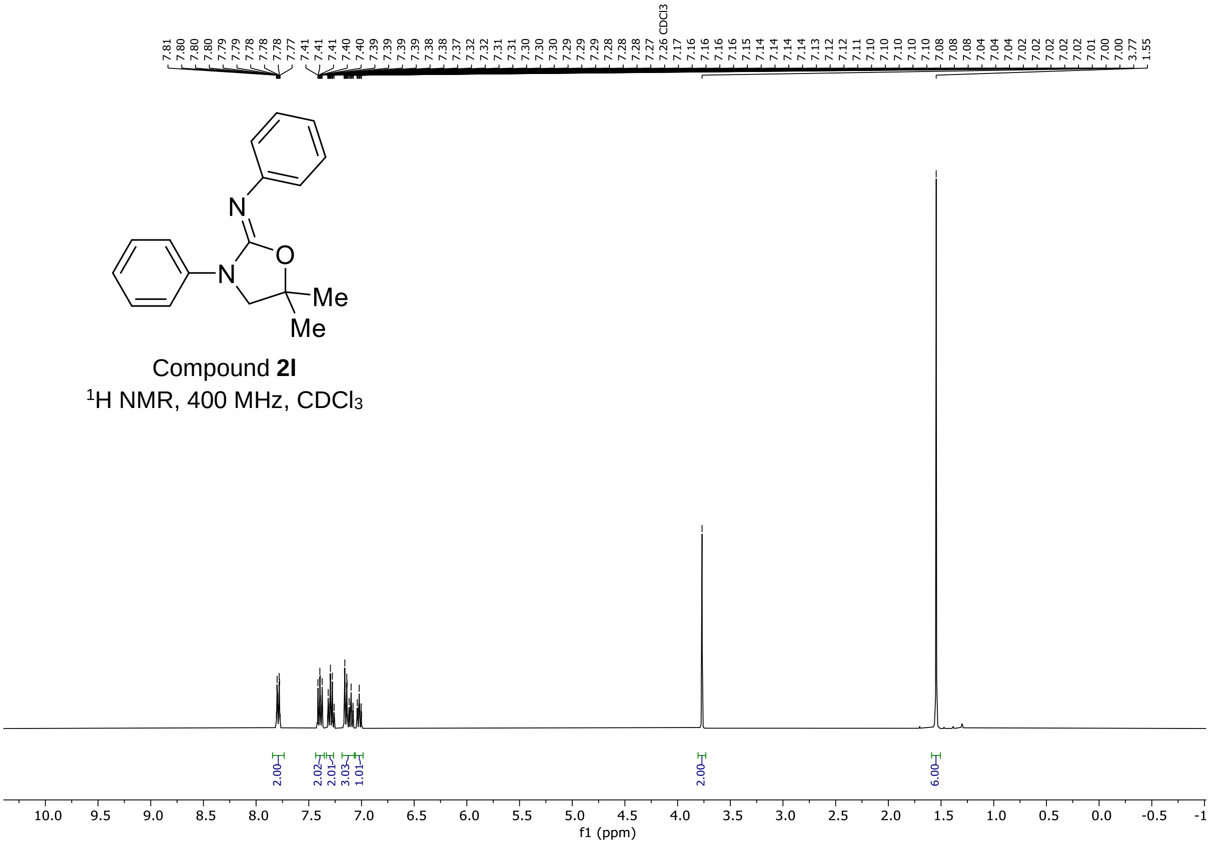
7.81 7.80 7.80 7.79 7.79 7.78 7.78 7.78 7.77 7.41 7.41 7.41 7.40 7.39 7.39 7.39 7.39 7.38 7.38 7.37 7.32 7.32 7.31 7.31 7.30 7.30 7.30 7.29 7.29 7.29 7.28 7.28 7.28 7.27 7.26 CDCl3 7.17 7.16 7.16 7.16 7.15 7.14 7.14 7.14 7.14 7.13 7.12 7.12 7.11 7.10 7.10 7.10 7.10 7.10 7.08 7.08 7.08 7.04 7.04 7.02 7.02 7.02 7.02 7.01 7.00 3.77 1.55
N
O
N
Me
Me
Compound **2l**
^{1}H NMR, 400 MHz, $CDCl_3$
2.00
2.02
2.01
3.03
1.01
2.00
6.00
f1 (ppm)

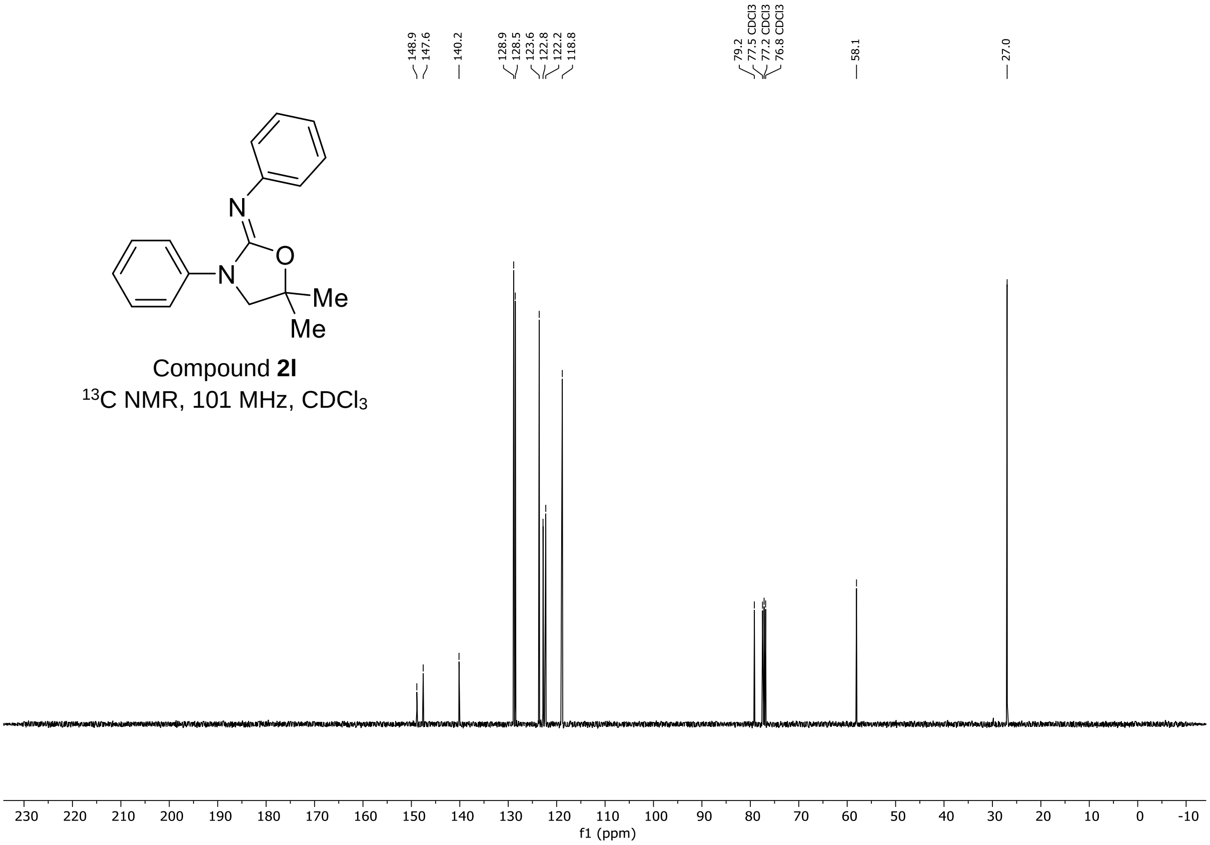
148.9 147.6 140.2 128.9 128.5 123.6 122.8 122.2 118.8 79.2 77.5 CDCl3 77.2 CDCl3 76.8 CDCl3 58.1 27.0
N
O
N
Me
Me
Compound **2l**
^{13}C NMR, 101 MHz, $CDCl_3$
f1 (ppm)

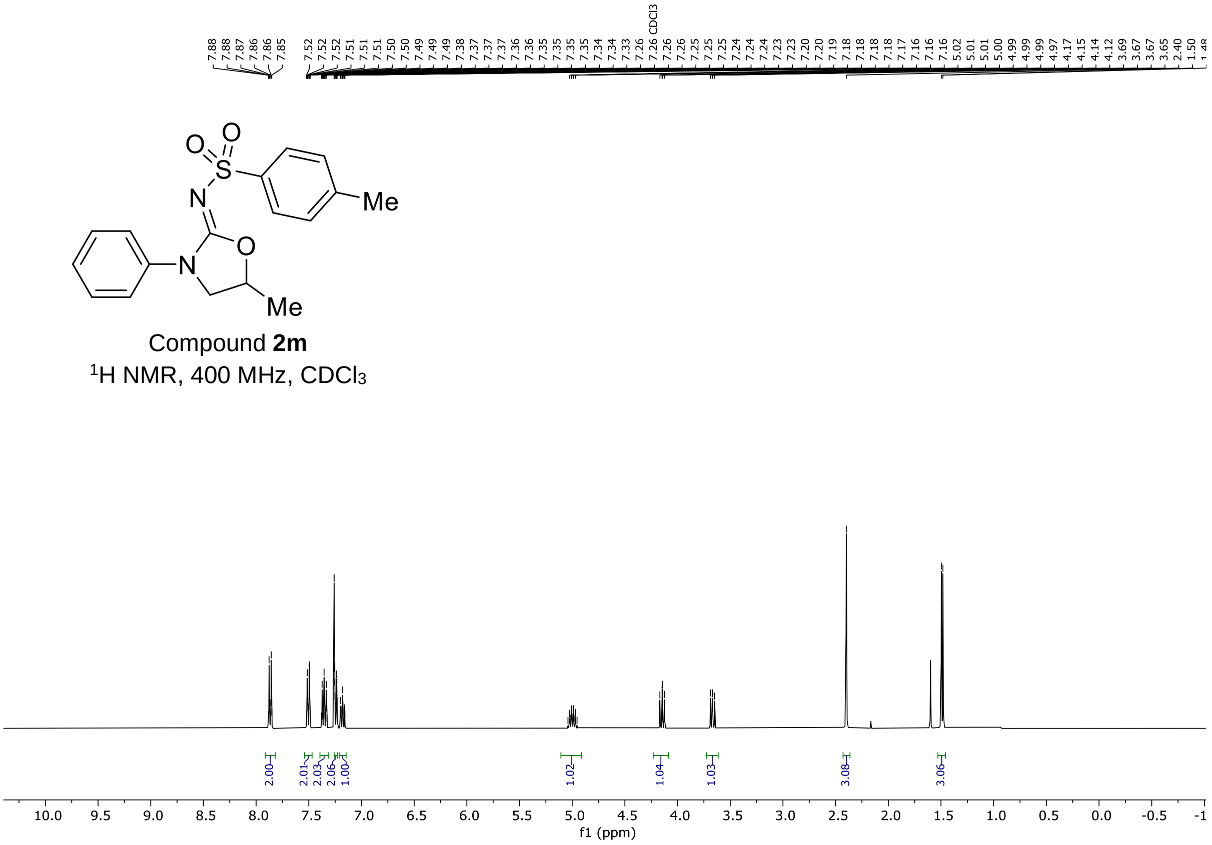
Me
Me
Compound **2m**
^{1}H NMR, 400 MHz, CDCl$_3$
f1 (ppm)

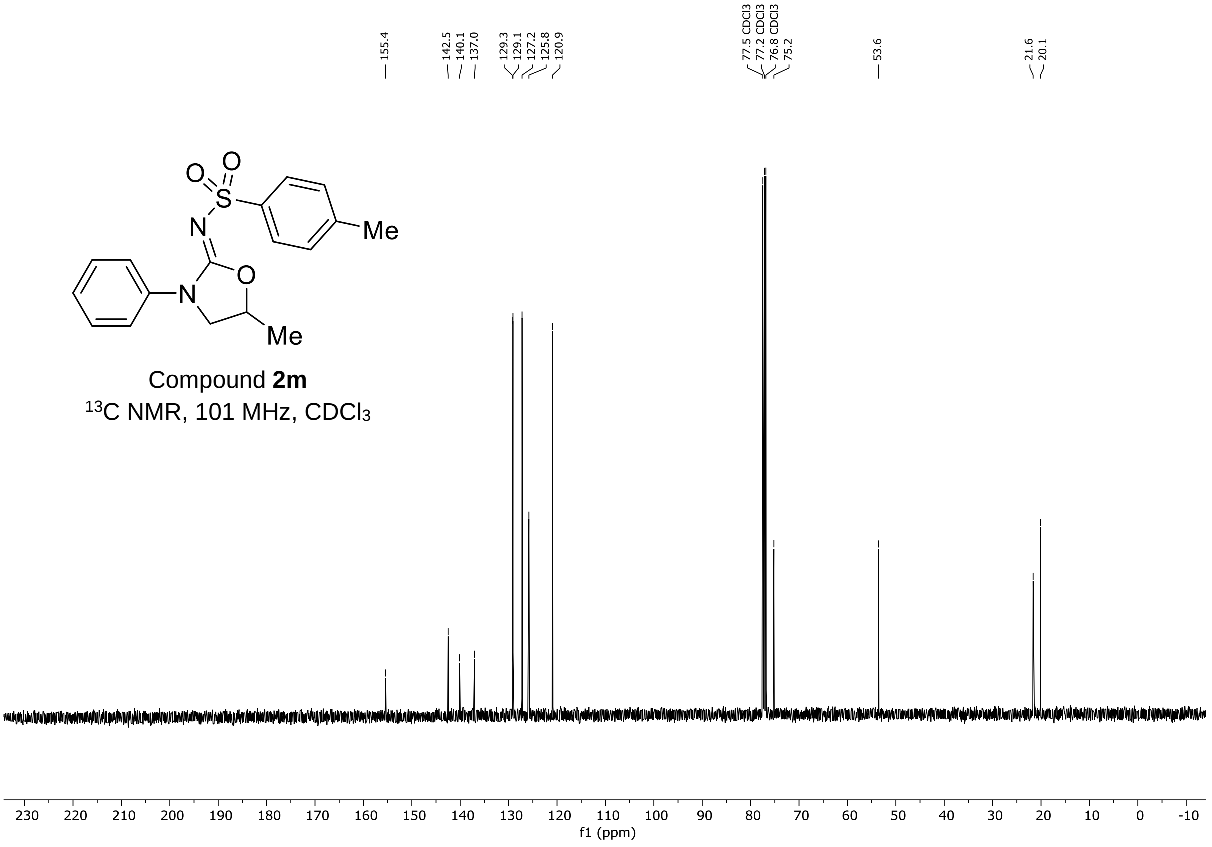
155.4
142.5
140.1
137.0
129.3
129.1
127.2
125.8
120.9
77.5 CDCl3
77.2 CDCl3
76.8 CDCl3
75.2
53.6
21.6
20.1
Me
Me
Compound **2m**
^{13}C NMR, 101 MHz, CDCl$_3$
f1 (ppm)

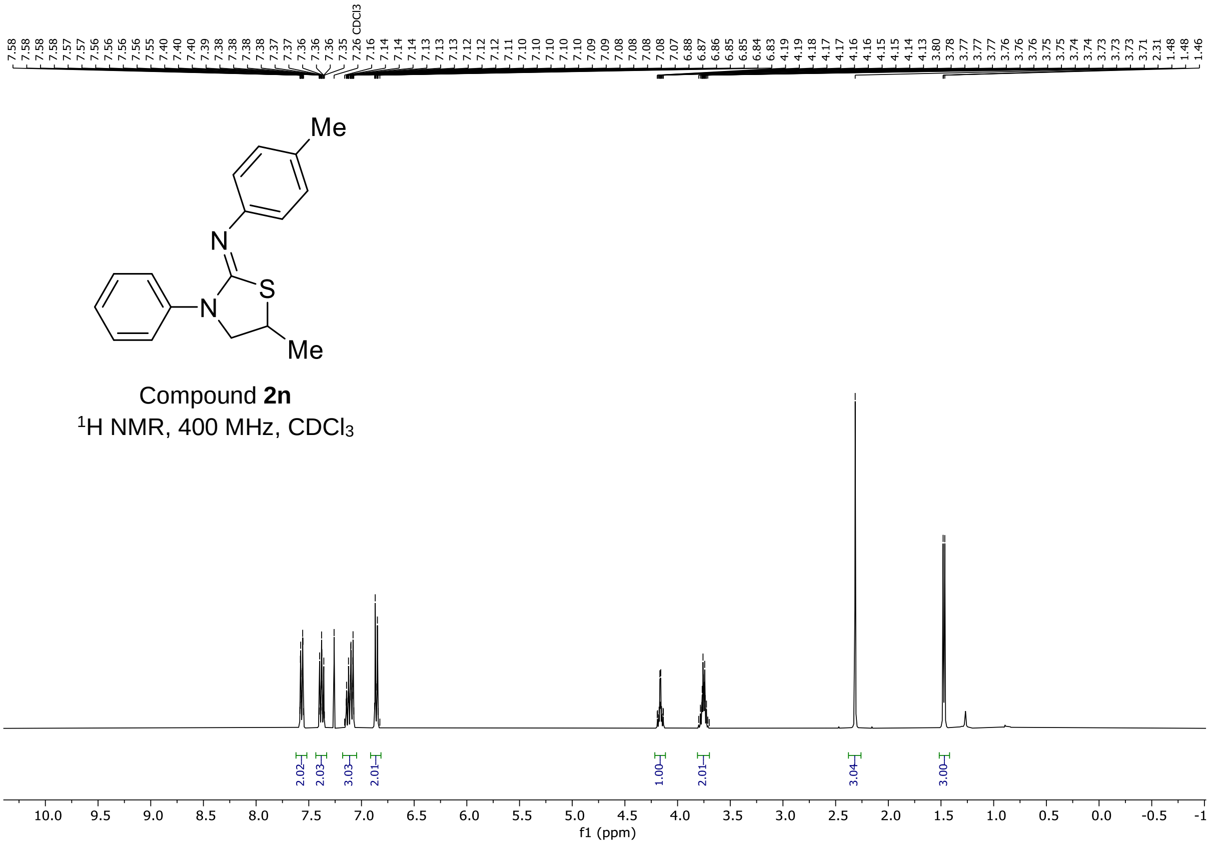
7.58 7.58 7.58 7.57 7.57 7.56 7.56 7.56 7.55 7.40 7.40 7.40 7.39 7.38 7.38 7.38 7.38 7.37 7.37 7.36 7.36 7.36 7.35 7.26 CDCl3 7.16 7.14 7.14 7.14 7.13 7.13 7.13 7.13 7.12 7.12 7.12 7.11 7.10 7.10 7.10 7.10 7.10 7.09 7.09 7.08 7.08 7.08 7.08 7.07 6.88 6.87 6.86 6.85 6.85 6.84 6.83 4.19 4.19 4.18 4.17 4.17 4.16 4.16 4.15 4.15 4.14 4.13 3.80 3.78 3.77 3.77 3.77 3.76 3.76 3.75 3.75 3.74 3.74 3.73 3.73 3.73 3.71 2.31 1.48 1.48 1.46
Me
N
S
N
Me
Compound 2n
1H NMR, 400 MHz, CDCl3
2.02 2.03 3.03 2.01 1.00 2.01 3.04 3.00
10.0 9.5 9.0 8.5 8.0 7.5 7.0 6.5 6.0 5.5 5.0 4.5 4.0 3.5 3.0 2.5 2.0 1.5 1.0 0.5 0.0 -0.5 -1
f1 (ppm)

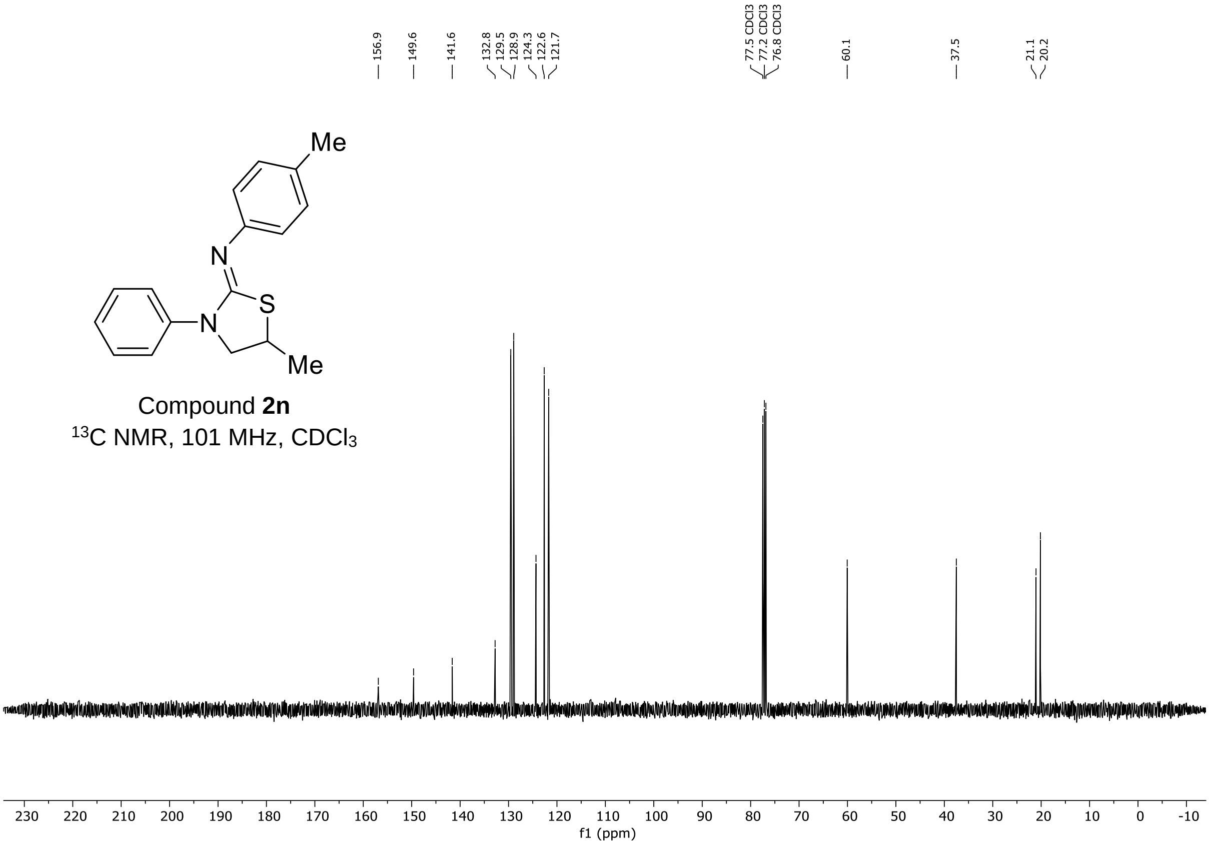
156.9 149.6 141.6 132.8 129.5 128.9 124.3 122.6 121.7 77.5 CDCl3 77.2 CDCl3 76.8 CDCl3 60.1 37.5 21.1 20.2
Me
N
S
N
Me
Compound 2n
13C NMR, 101 MHz, CDCl3
230 220 210 200 190 180 170 160 150 140 130 120 110 100 90 80 70 60 50 40 30 20 10 0 -10
f1 (ppm)

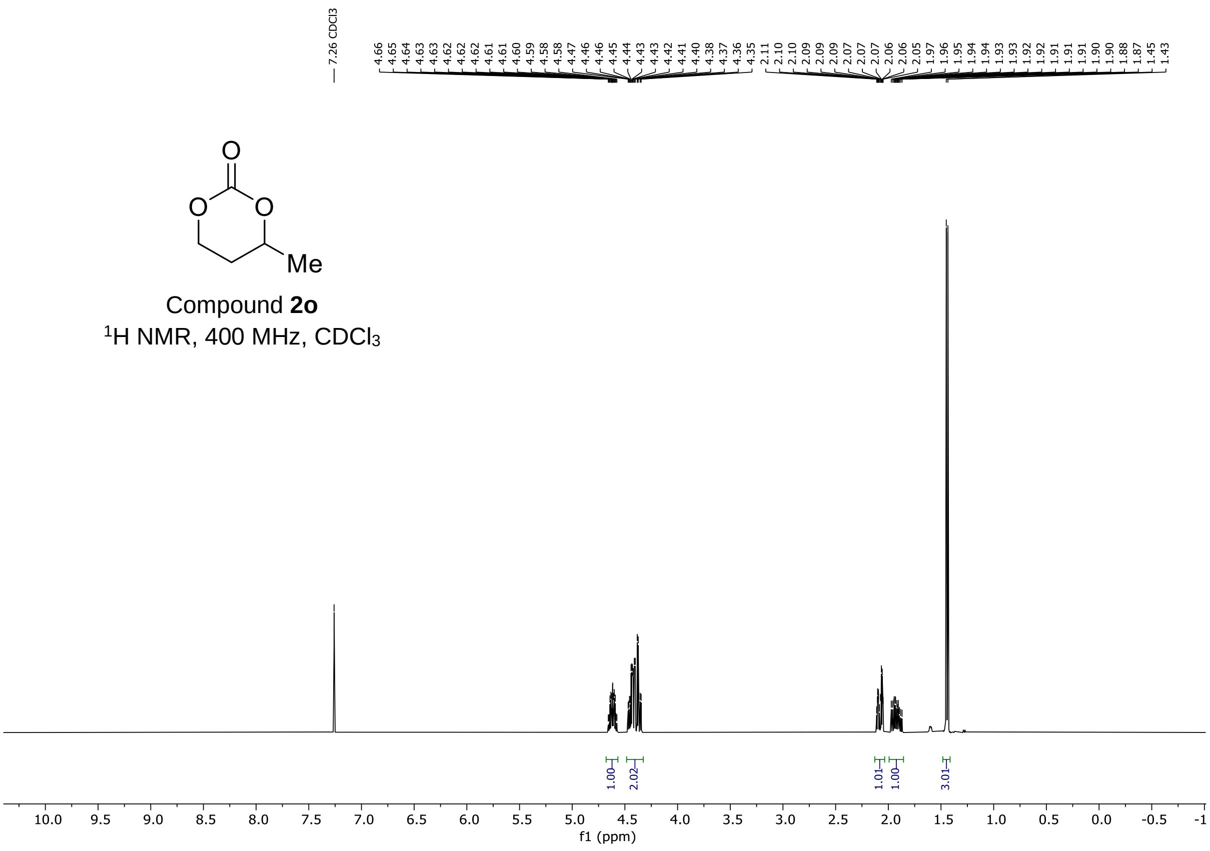

7.26 CDCl3
4.66 4.65 4.64 4.63 4.63 4.62 4.62 4.62 4.61 4.61 4.60 4.59 4.58 4.58 4.47 4.46 4.46 4.45 4.44 4.43 4.43 4.42 4.41 4.40 4.38 4.37 4.36 4.35
2.11 2.10 2.09 2.09 2.09 2.07 2.07 2.06 2.06 2.05 1.97 1.96 1.95 1.94 1.94 1.93 1.93 1.92 1.92 1.91 1.91 1.91 1.90 1.90 1.88 1.87 1.45 1.43
O
O
O
Me
Compound 2o
1H NMR, 400 MHz, CDCl3
1.00
2.02
1.01
1.00
3.01
10.0 9.5 9.0 8.5 8.0 7.5 7.0 6.5 6.0 5.5 5.0 4.5 4.0 3.5 3.0 2.5 2.0 1.5 1.0 0.5 0.0 -0.5 -1
f1 (ppm)

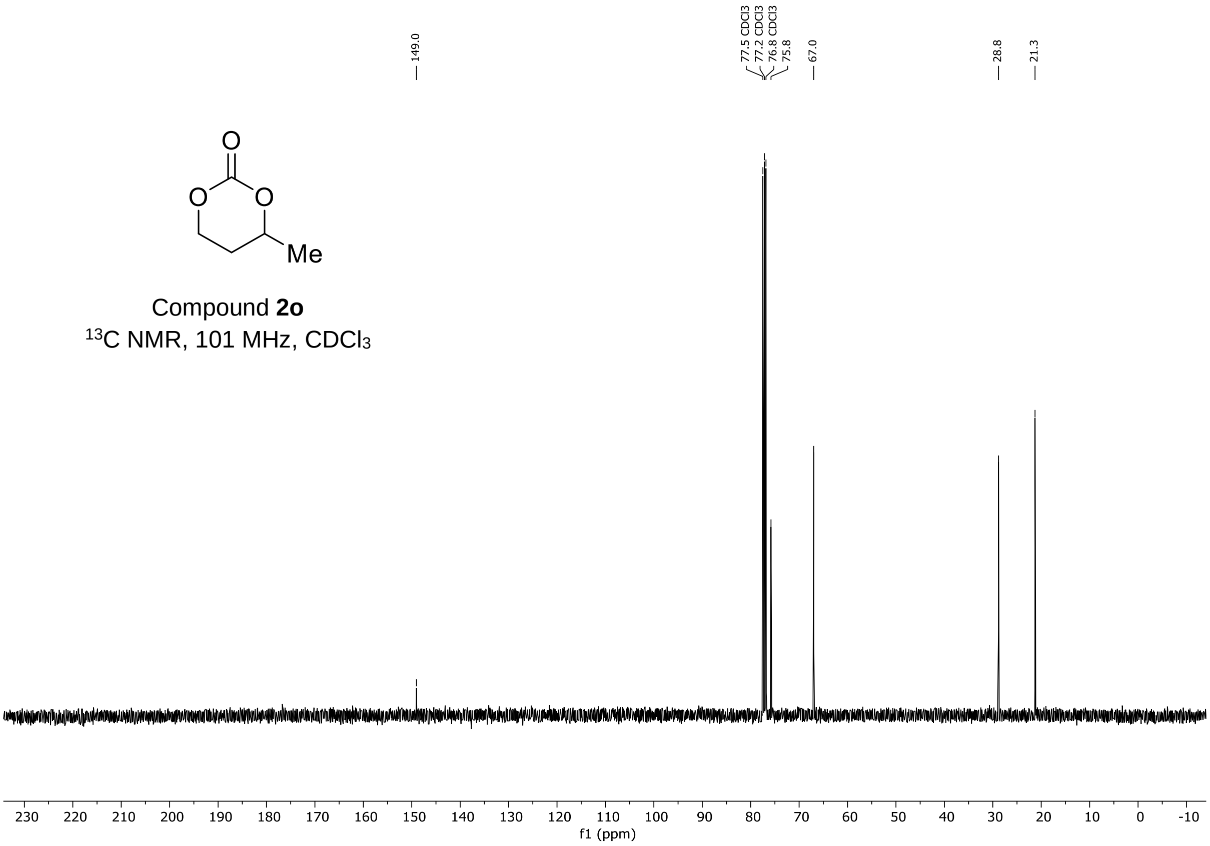

149.0
77.5 CDCl3
77.2 CDCl3
76.8 CDCl3
75.8
67.0
28.8
21.3
O
O
O
Me
Compound 2o
13C NMR, 101 MHz, CDCl3
230 220 210 200 190 180 170 160 150 140 130 120 110 100 90 80 70 60 50 40 30 20 10 0 -10
f1 (ppm)

Compound **2p**
^{1}H NMR, 400 MHz, $CDCl_3$

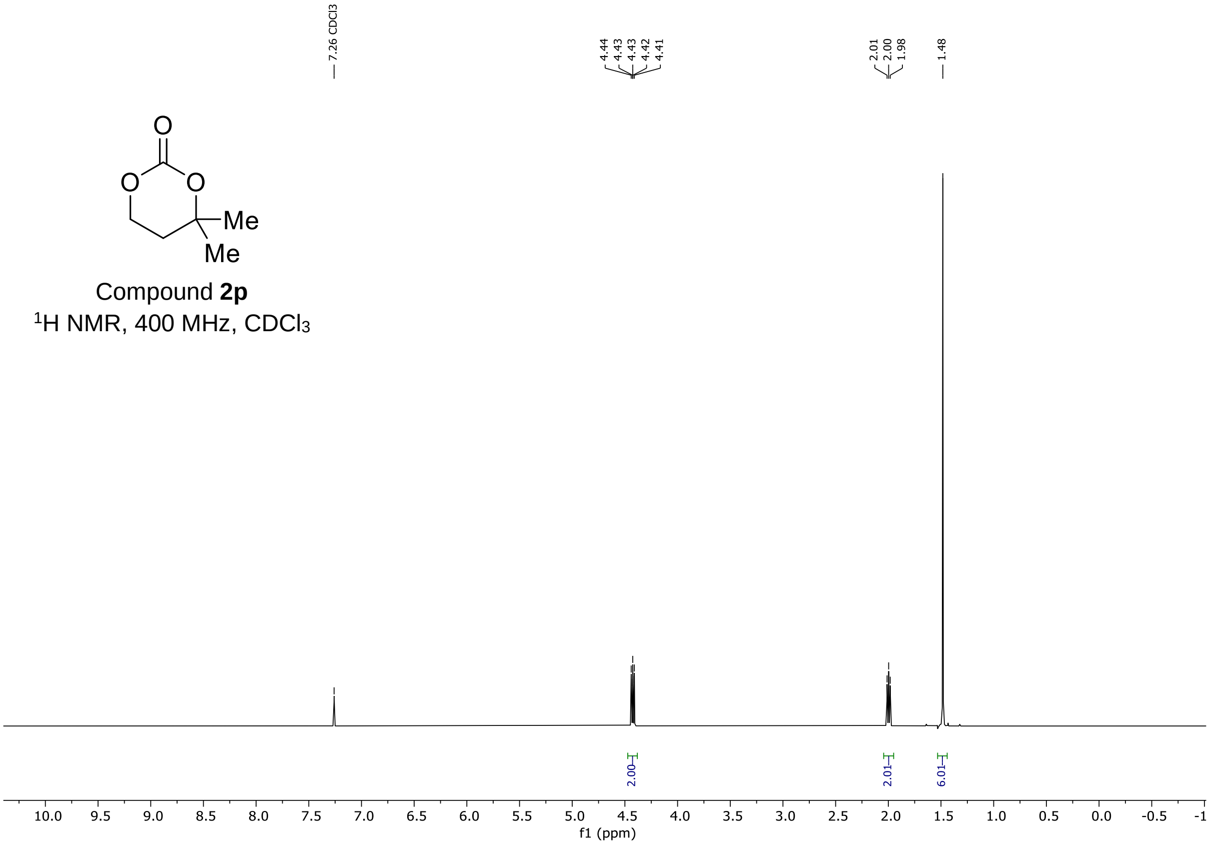

Compound **2p**
^{13}C NMR, 101 MHz, $CDCl_3$

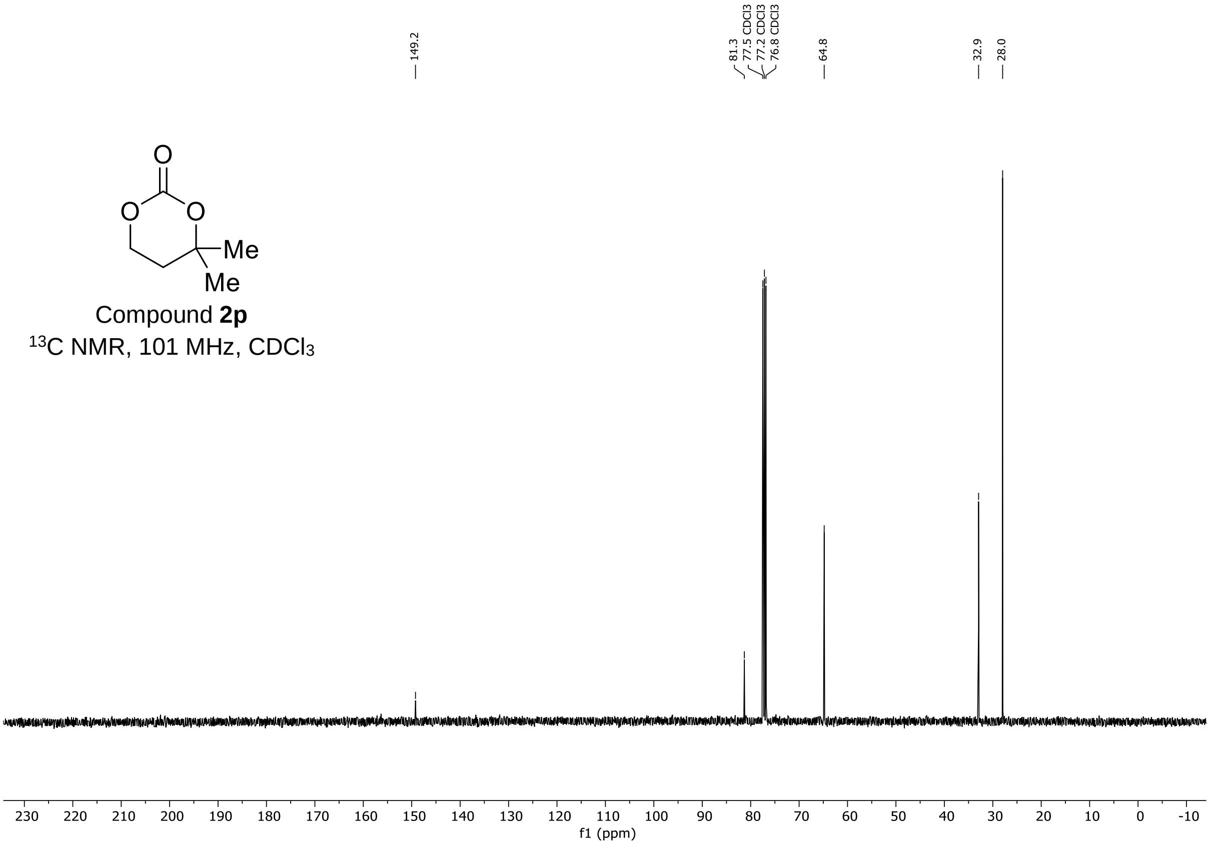

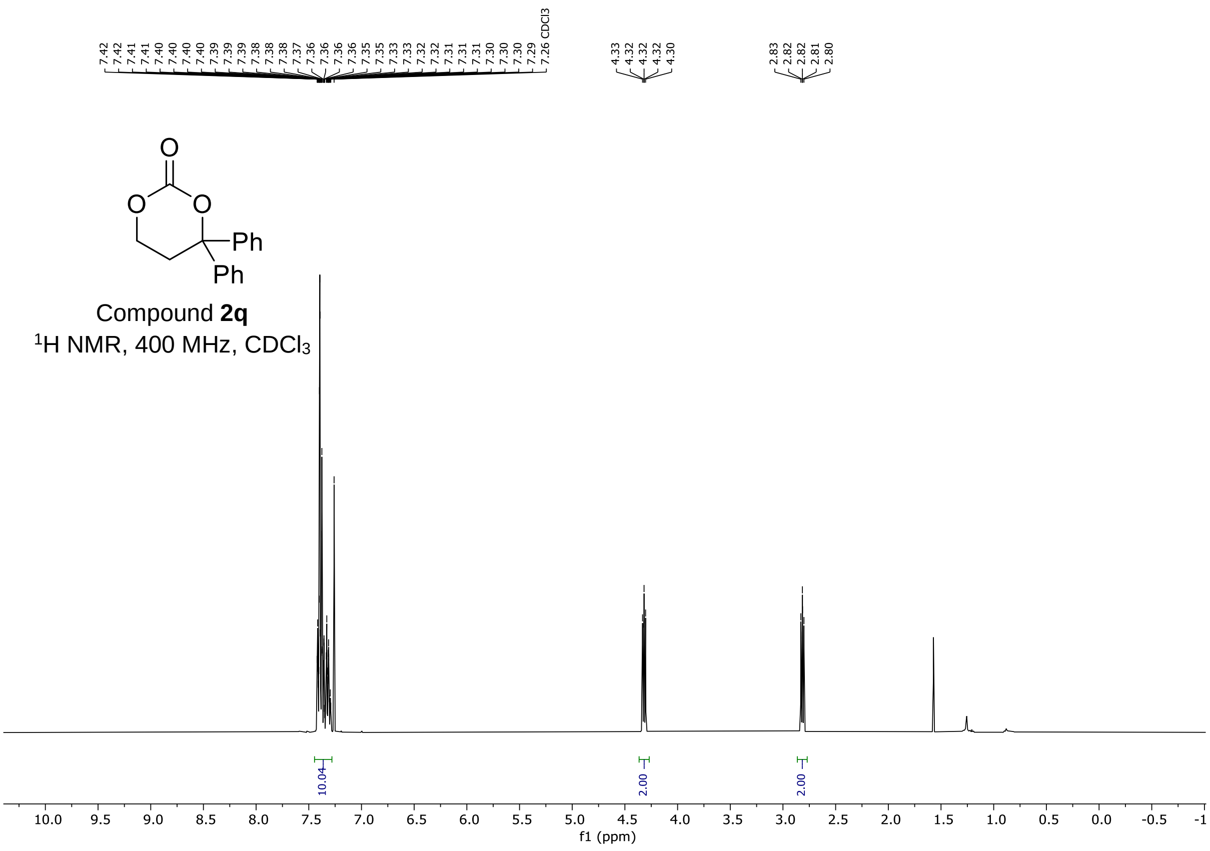
Ph
Ph
Compound **2q**
^{1}H NMR, 400 MHz, $CDCl_3$
10.04
2.00
2.00
f1 (ppm)

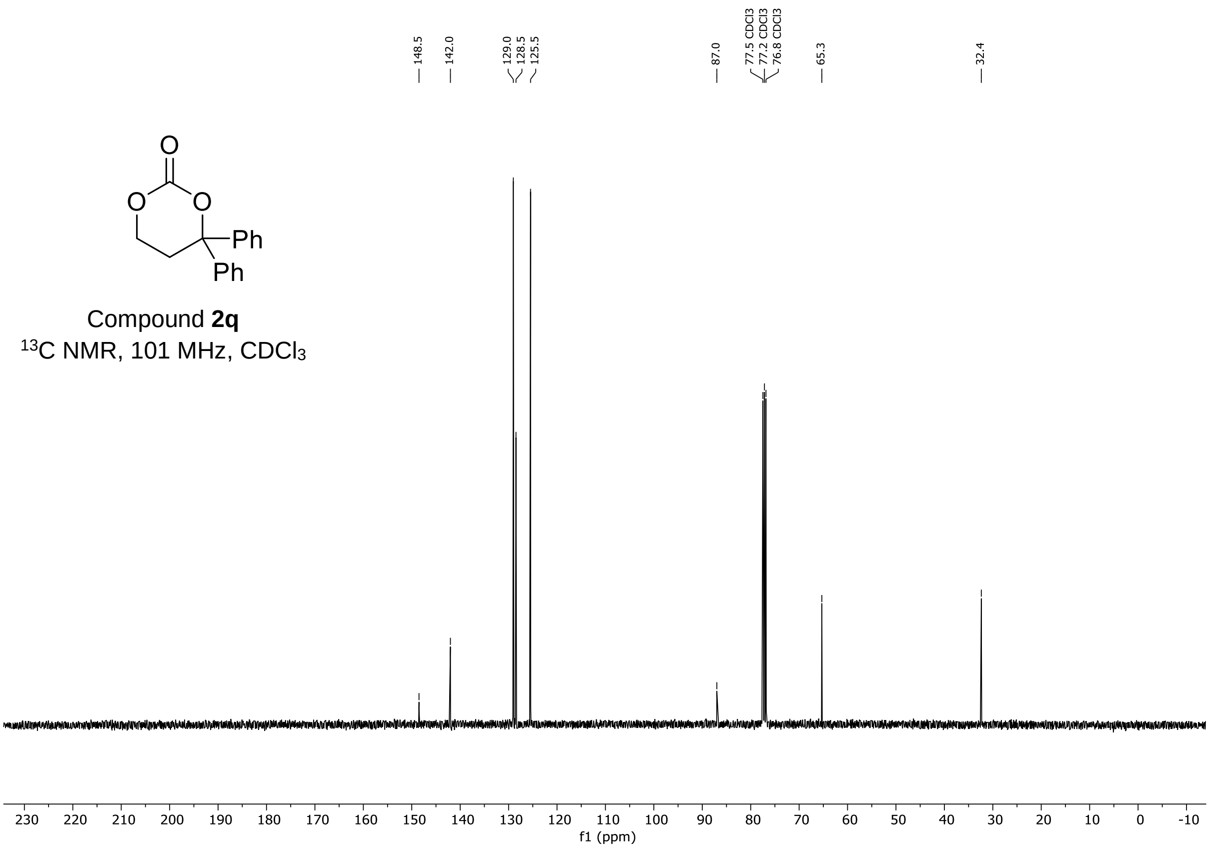
148.5
142.0
129.0
128.5
125.5
87.0
77.5 CDCl3
77.2 CDCl3
76.8 CDCl3
65.3
32.4
Ph
Ph
Compound **2q**
^{13}C NMR, 101 MHz, $CDCl_3$
f1 (ppm)

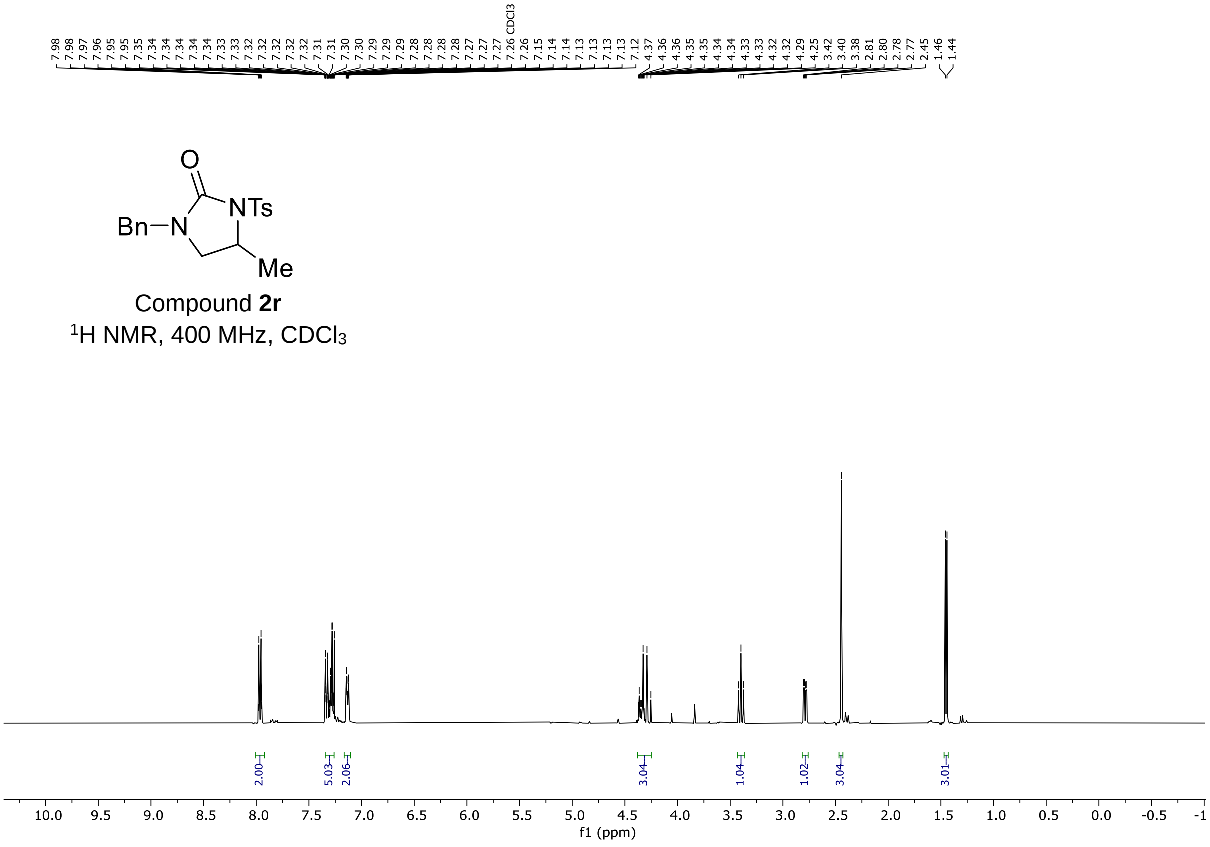
O
NTs
Bn—N
Me
Compound **2r**
^{1}H NMR, 400 MHz, $CDCl_3$
2.00
5.03
2.06
3.04
1.04
1.02
3.04
3.01
f1 (ppm)

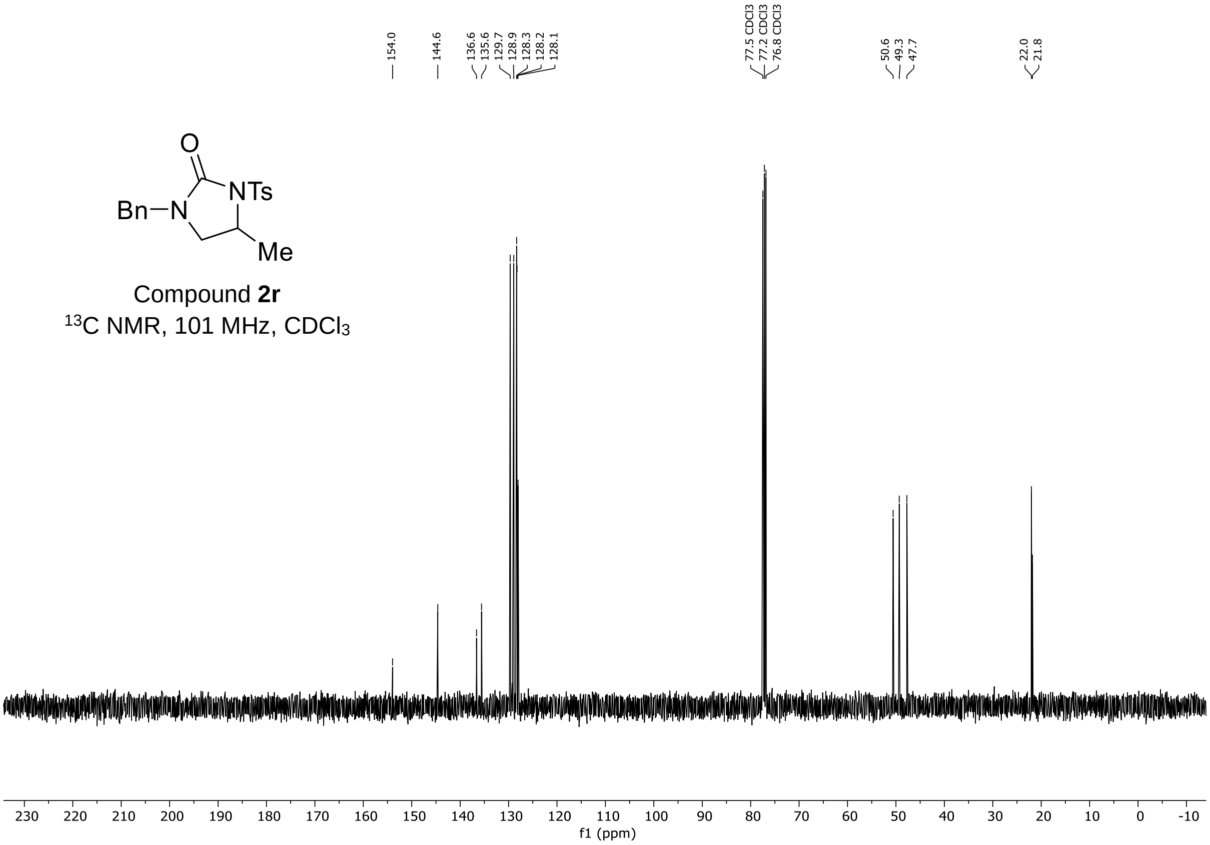
154.0
144.6
136.6
135.6
129.7
128.9
128.3
128.2
128.1
77.5 CDCl3
77.2 CDCl3
76.8 CDCl3
50.6
49.3
47.7
22.0
21.8
O
NTs
Bn—N
Me
Compound **2r**
^{13}C NMR, 101 MHz, $CDCl_3$
f1 (ppm)

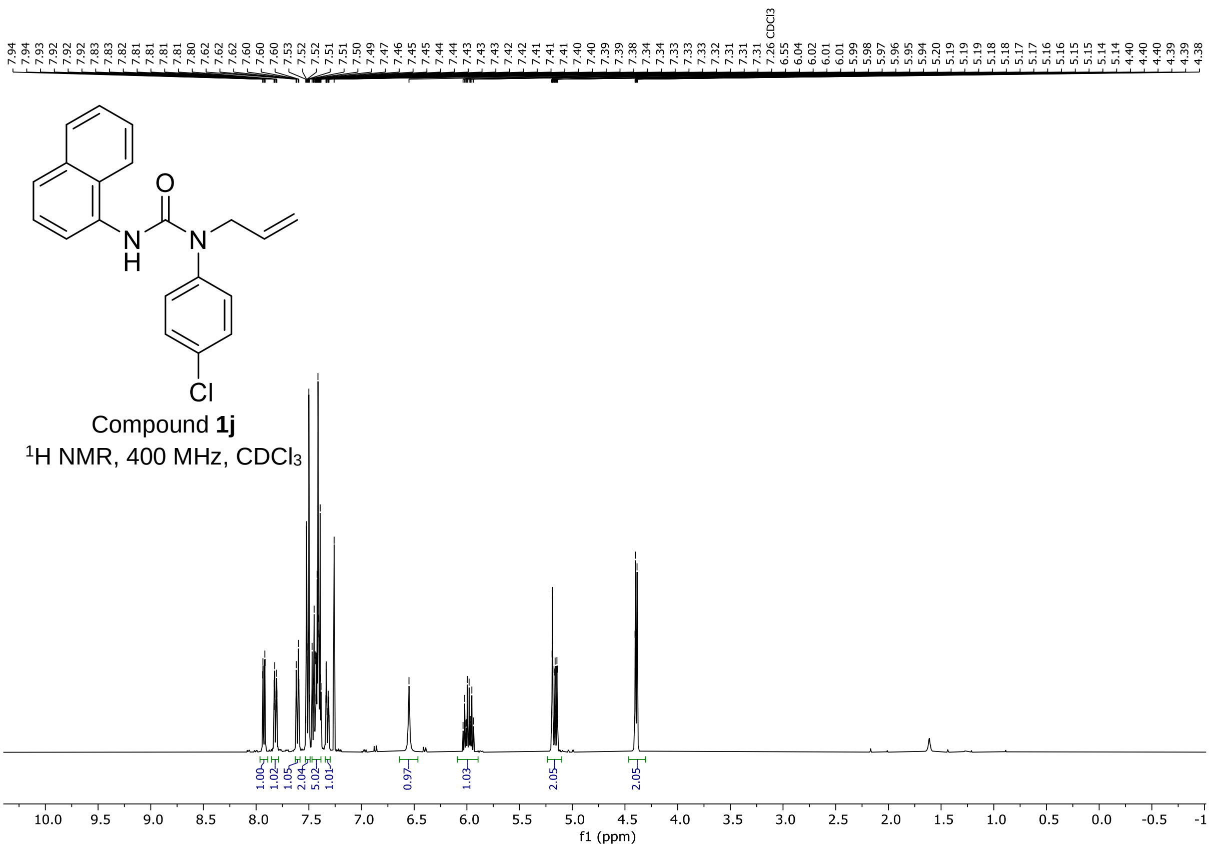

Compound **1j**
^{1}H NMR, 400 MHz, $CDCl_3$
f1 (ppm)

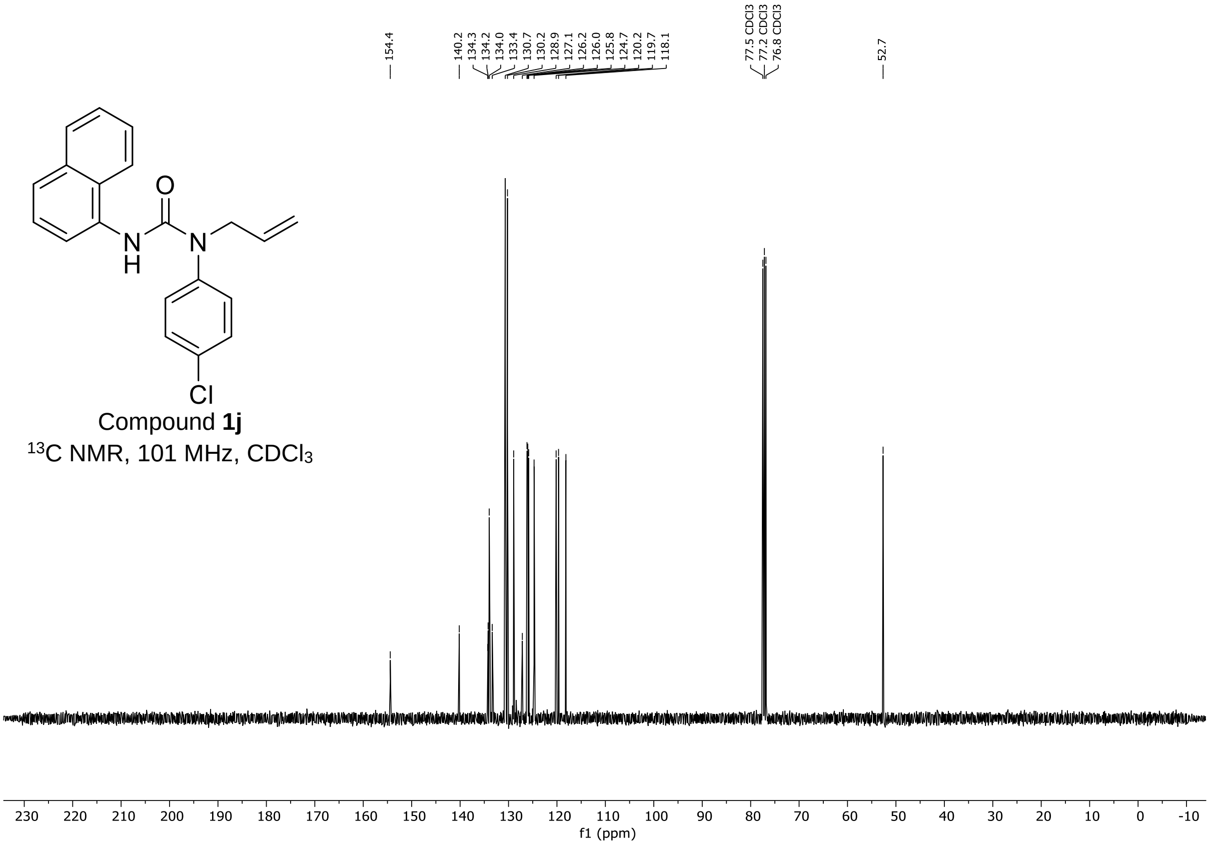

154.4
140.2
134.3
134.2
134.0
133.4
130.7
130.2
128.9
127.1
126.2
126.0
125.8
124.7
120.2
119.7
118.1
77.5 CDCl3
77.2 CDCl3
76.8 CDCl3
52.7
Compound **1j**
^{13}C NMR, 101 MHz, $CDCl_3$
f1 (ppm)

6. References

(1) Rössler, S. L.; Jelier, B. J.; Tripet, P. F.; Shemet, A.; Jeschke, G.; Togni, A.; Carreira, E. M. Pyridyl Radical Cation for C−H Amination of Arenes. *Angew. Chem. Int. Ed.* **2019**, *58*, 526-531.

(2) Fischer, D. M.; Lindner, H.; Amberg, W. M.; Carreira, E. M. Intermolecular Organophotocatalytic Cyclopropanation of Unactivated Olefins. *J. Am. Chem. Soc.* **2023**, *145*, 774-780.

(3) Nagai, T.; Mimata, N.; Terada, Y.; Sebe, C.; Shigehisa, H. Catalytic Dealkylative Synthesis of Cyclic Carbamates and Ureas via Hydrogen Atom Transfer and Radical-Polar Crossover. *Org. Lett.* **2020**, *22*, 5522-5527.

(4) (a) Hopkins, B. A.; Wolfe, J. P. Synthesis of Enantiomerically Enriched Imidazolidin-2-Ones through Asymmetric Palladium-Catalyzed Alkene Carboamination Reactions. *Angew. Chem. Int. Ed.* **2012**, *51*, 9886-9890. (b) Yip, K.-T.; Yang, D. Pd(II)-Catalyzed Intramolecular Amidoarylation of Alkenes with Molecular Oxygen as Sole Oxidant. *Org. Lett.* **2011**, *13*, 2134-2137. (c) Ahmed, N.; Khatoon, S. Facile Electrochemical Intramolecular Amination of Urea-Tethered Terminal Alkenes for the Synthesis of Cyclic Ureas. *ChemistryOpen* **2018**, *7*, 576-582.

(5) Islam, M.; Kariuki, B. M.; Shafiq, Z.; Wirth, T.; Ahmed, N. Efficient Electrosynthesis of Thiazolidin-2-imines via Oxysulfurization of Thiourea-Tethered Terminal Alkenes Using the Flow Microreactor. *Eur. J. Org. Chem.* **2019**, *2019*, 1371-1376.

(6) Ohuchi, S.; Koyama, H.; Shigehisa, H. Catalytic Synthesis of Cyclic Guanidines via Hydrogen Atom Transfer and Radical-Polar Crossover. *ACS Catal.* **2021**, *11*, 900-906.

(7) (a) Malkov, A. V.; Derrien, N.; Barłóg, M.; Kočovský, P. Palladium-Catalyzed Alkoxycarbonylation of Terminal Alkenes To Produce α,β-Unsaturated Esters: The Key Role of Acetonitrile as a Ligand. *Chem. Eur. J* **2014**, *20*, 4542-4547. (b) Ruchti, J.; Carreira, E. M. Ir-Catalyzed Reverse Prenylation of 3-Substituted Indoles: Total Synthesis of (+)-Aszonalenin and (−)-Brevicompanine B. *J. Am. Chem. Soc.* **2014**, *136*, 16756-16759. (c) Maquilón, C.; Della Monica, F.; Limburg, B.; Kleij, A. W. Photocatalytic Synthesis of Substituted Cyclic Carbonate Monomers for Ring-Opening Polymerization. *Adv. Synth. Catal.* **2021**, *363*, 4033-4040.

(8) Lindner, H.; Amberg, W. M.; Martini, T.; Fischer, D. M.; Moore, E.; Carreira, E. M. Photo- and Cobalt-Catalyzed Synthesis of Heterocycles via Cycloisomerization of Unactivated Olefins. *Angew. Chem. Int. Ed.* **2024**, *63*, e202319515.
